# Raman, UV–Vis Absorption, and Fluorescence Spectroelectrochemistry for Studying the Enhancement of the Raman Scattering Using Nanocrystals Activated by Metal Cations

**DOI:** 10.1021/acs.analchem.3c01172

**Published:** 2023-10-23

**Authors:** Sheila Hernandez, Martin Perez-Estebanez, William Cheuquepan, Juan V. Perales-Rondon, Aranzazu Heras, Alvaro Colina

**Affiliations:** † Department of Chemistry, Universidad de Burgos, Pza. Misael Bañuelos s/n, E-09001 Burgos, Spain; ‡ Bernal Institute, University of Limerick (UL), Limerick V94 T9PX, Ireland; § Department of Chemical Sciences, School of Natural Sciences, University of Limerick (UL), Limerick V94 T9PX, Ireland

## Abstract

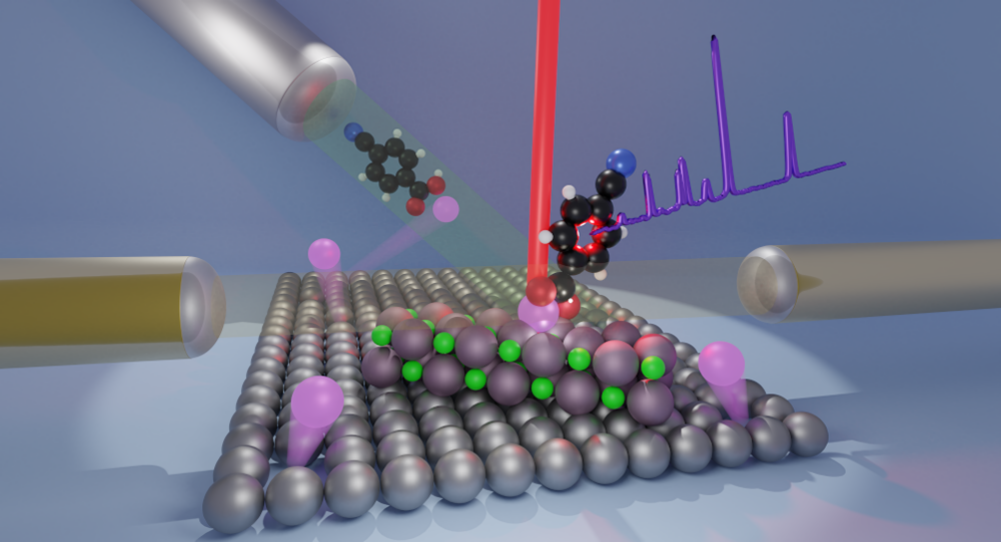

Raman signal enhancement is fundamental to develop different analytical tools for chemical analysis, interface reaction studies, or new materials characterization, among others. Thus, phenomena such as surface-enhanced Raman scattering (SERS) have been used for decades to increase the sensitivity of Raman spectroscopy, leading to a huge development of this field. Recently, an alternative method to SERS for the amplification of Raman signals has been reported. This method, known as electrochemical surface oxidation-enhanced Raman scattering (EC-SOERS), has been experimentally described. However, to date, it has not yet been fully understood. In this work, new experimental data that clarify the origin of the Raman enhancement in SOERS are provided. The use of a complete and unique set of combined spectroelectrochemistry techniques, including time-resolved *operando* UV–vis absorption, fluorescence, and Raman spectroelectrochemistry, reveals that such enhancement is related to the generation of dielectric or semiconductor nanocrystals on the surface of the electrode and that the interaction between the target molecule and the dielectric substrate is mediated by metal cations. According to these results, the interaction metal electrode–nanocrystal–metal cation–molecule is proposed as being responsible for the Raman enhancement in Ag and Cu substrates. Elucidation of the origin of the Raman enhancement will help to promote the rational design of SOERS substrates as an attractive alternative to the well-known SERS phenomenon.

## Introduction

Raman signal enhancement is essential to increasing the low sensitivity of Raman spectroscopy. There are different strategies to amplify this signal, with surface-enhanced Raman scattering (SERS) being the most widely used.^1−7^ Some other enhancement strategies derived from SERS, such as tip-enhanced Raman spectroscopy (TERS)^8−11^ or shell-isolated nanoparticle enhanced Raman spectroscopy (SHINERS),^12−14^ are attracting the interest of researchers because of the high spatial resolution and the capability of working in surface science without the influence of the SERS substrate. The role of halides on SERS performance has been studied since the discovery of SERS, being well discussed by several groups in the past decades.^15−17^ Halides play a role not only in the generation of the SERS substrate but also in the adsorption of the target molecule, as a mediator in these processes.^18,19^ For this reason, our research group studied very different conditions, modulating the halide concentration and pH to improve the performance of EC-SERS detection. While these experiments were performed, an unexpected enhancement of the Raman signal was observed during the oxidation of a silver electrode under specific electrolytic conditions.

In 2018, our research group reported for the first time the electrochemical surface oxidation-enhanced Raman scattering (EC-SOERS) phenomenon.^20^ Briefly, while generating a SERS substrate, the amplification of the Raman signal was observed during the electrochemical oxidation of a silver electrode in the presence of a low concentration of chloride in acidic medium.^20^ At that moment, the origin of this enhancement has not yet been explained since the presence of plasmonic metal structures is not expected at anodic potentials.^7,21^ In fact, the enhancement of the Raman signal was not only observed at these anodic potentials but also disappeared when the electrochemical system was left at open circuit potential or when cathodic potentials were applied.^20^ Due to the complex nature of the phenomenon, the use of *operando* time-resolved Raman spectroelectrochemistry (TR-Raman-SEC) was mandatory to understand EC-SOERS. Nevertheless, although the origin of the Raman enhancement was not fully explained, it was suggested that silver chloride crystals generated on the electrode surface should be involved in the Raman signal enhancement.^20^

So far, EC-SOERS has led to very interesting results in chemical analysis,^22,23^ demonstrating high sensitivity^24^ and chemical selectivity,^25^ allowing quantitative analysis, with good reproducibility (RSD% ≤ 10%) even in complex matrices.^22,23^ EC-SOERS can also be combined with EC-SERS^26,27^ to increase the spectroscopic information in two different ways: using EC-SERS or EC-SOERS to selectively analyze different molecular structures^26^ or obtaining a double fingerprint of the same analyte, leading to significant differences between EC-SERS and EC-SOERS spectra recorded at different potentials in the same experiment.^27^

**Figure 1:**
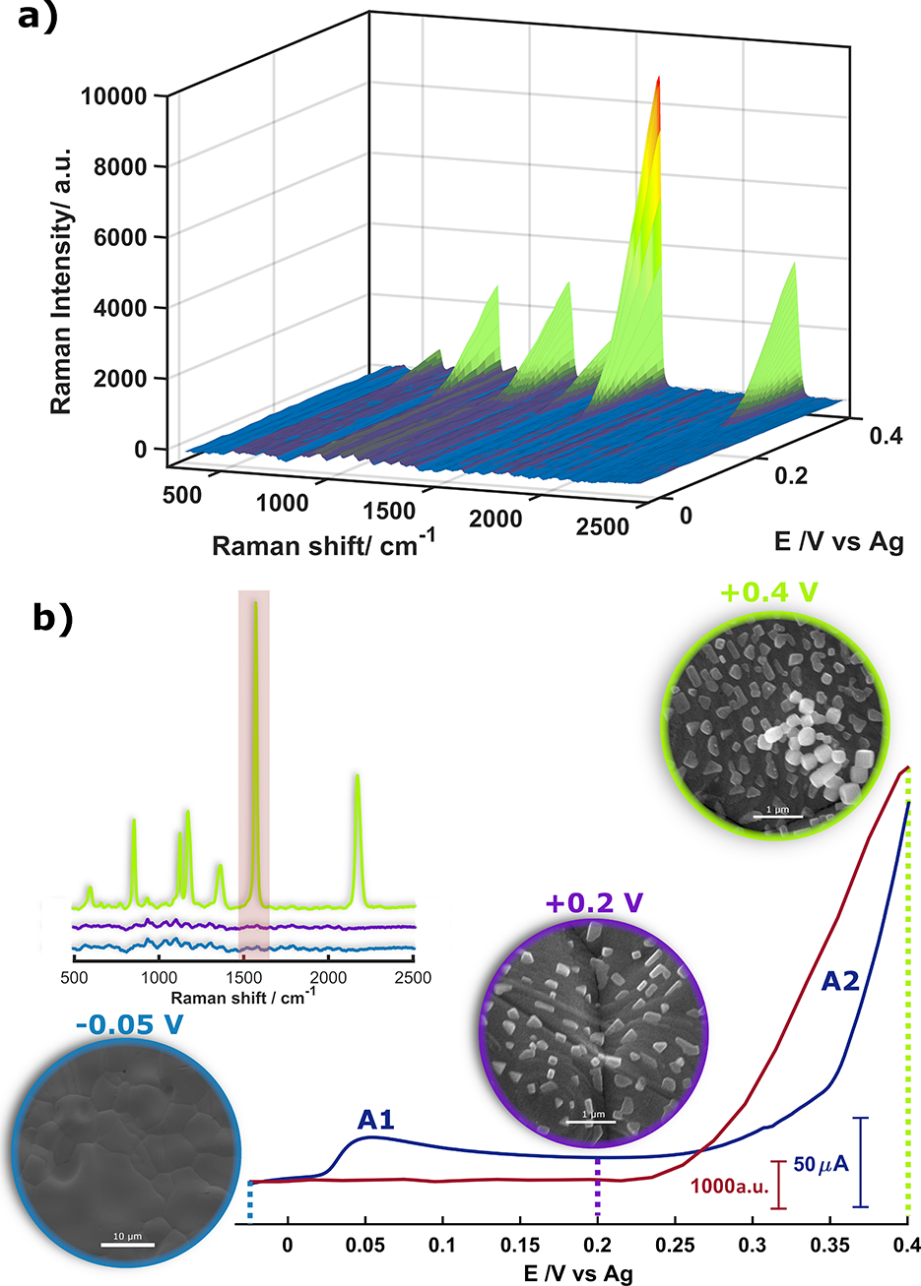
(a) 3D plot of Raman spectral evolution of 4-cyanobenzoic acid during the oxidation of a silver electrode. (b) LSV (blue line) and Raman intensity at 1539 cm^–1^ (garnet line) during the oxidation of the electrode. SEM images and Raman spectra at different potentials of the experiment (inset). Electrochemical conditions: 0.1 mM 4-cyanobenzoic acid + 5 mM KCl + 0.1 M HClO_4_. LSV was performed between −0.025 and +0.40 V. Scan rate: 20 mV s^–1^.

A general view of EC-SOERS is illustrated in Figure 1a, which shows a 3D plot of the EC-SOERS enhancement for 4-cyanobenzoic acid in the presence of chloride using a silver electrode during a TR-Raman-SEC experiment. From this figure, the enhancement of the Raman signal can be clearly observed at potentials where silver is being oxidized. Focusing on the voltammetric signal, a metal electrode is oxidized in the presence of a precipitating agent to first generate crystals of the corresponding salt on the electrode surface (A1, blue line in Figure 1b) and later release metal cations from the electrode (A2, blue line in Figure 1b). During the release of metal cations, the Raman signal of the target molecule is amplified (garnet line, Figure 1b), obtaining analytical enhancement factors (AEF) greater than 10^5^ for 4-cyanobenzoic acid and other molecules.^24^ Insets in Figure 1b show the SEM images of the electrode surface at different applied potentials, showing the generation of nanocrystals on the silver surface during the experiment.

Although EC-SOERS has driven several applications in the field of chemical analysis, the origin of the Raman enhancement needs to be elucidated to promote the rational design of EC-SOERS substrates.

The high AEFs obtained in a previous study^24^ indicate that the amplification must be due to an electromagnetic mechanism, as well as to a chemical mechanism, similar to SERS. However, the experimental conditions in which EC-SOERS is observed (anodic potentials and acidic media) rule out the presence of plasmonic Ag(0) structures.^7,21^ An alternative to the classical SERS of plasmonic metal nanostructures can be found in the amplification of Raman scattering with dielectrics and semiconductors.^28−31^ Dielectric/semiconductor-based SERS substrates have been overshadowed by plasmonic metals for many years. Recent works developed by several researchers and the theoretical understanding provided by Lombardi et al.^28,29^ have allowed the massive development of SERS substrates based on such materials.^30−35^ One of the main advantages of this SERS strategy is the tunability of the substrate, which allows the modulation of its plasmonic properties,^29,34^ as well as other effects included in the electromagnetic enhancement such as total internal reflection phenomena, interference-enhanced Raman scattering (IERS), and/or the use of Mie resonances to concentrate the exciting light and efficient collection of the scattered light.^28,29^

To the best of our knowledge, a full explanation of EC-SOERS has not yet been provided. Therefore, the purpose of this work is to provide full insight into EC-SOERS, pointing out the key factors responsible for this Raman signal enhancement. With this aim in mind, a battery of combined spectroelectrochemistry (SEC) techniques has been used. Simultaneous SEC techniques^36,37^ provide independent and complementary information about the processes taking place on the electrode surface. The combination of Raman with UV–vis absorption and with fluorescence provides simultaneous information not only about the enhancement of the Raman signal but also about the structures and chemical processes occurring at the electrode interface. The combination of all of these techniques yields an unequivocal vision of the role of the different structures and compounds involved in the enhancement of the Raman signal.

In this work, we demonstrate that EC-SOERS can be considered as a new strategy to enhance the Raman signal of molecules. A wide range of substrates and electrochemical conditions, much more diverse than the previous reports on silver substrates using chloride^20,24,27,38^ and bromide,^23,25^ will be used to induce the enhancement of the Raman signal. We will study different target molecules to demonstrate the capabilities of the proposed strategy and, at the same time, understand the mechanism of amplification of the Raman signal. Finally, we demonstrate that the applied potential is not a key factor in the origin of EC-SOERS and we study the activation of the Raman enhancement by metallic cations present in solution.

## Experimental Section

### Reagents

Perchloric acid (HClO_4_, 60%, Sigma-Aldrich), potassium chloride (KCl, 99%, Acros Organics), potassium bromide (KBr, 99%, Acros Organics), potassium iodide (KI, >99%, Labkem), potassium thiocyanate (KSCN, 99%, VWR), potassium hexacyanoferrate(II) (K_4_Fe(CN)_6_, 99%, Acros Organics), 4-cyanobenzoic acid (99%, Acros Organics), clopyralid (>98%, Tokyo Chemical Industry), benzoic acid (>99.5%, Sigma-Aldrich), gallocyanine (90% dye, Sigma-Aldrich), caffeic acid (98%, Sigma-Aldrich), alizarin RS (>99.5, Acros Organics), isonicotinic acid (99%, Alfa Aesar), sodium fluorescein (for analysis, Panreac), riboflavin (for analysis, Sigma-Aldrich), phthalic acid (>99.5%, Sigma-Aldrich), silver perchlorate monohydrate (AgClO_4_, 99.9%, Alfa Aesar), and copper sulfate (CuSO_4_, for analysis, Merck) were used.

All reagents were used as received without further purification. All solutions were freshly prepared by using ultrapure water obtained from a Millipore DirectQ purification system provided by Millipore (18.2 MΩ cm resistivity at 25 °C).

### Raman SEC

*In situ* time-resolved Raman SEC was performed with a customized SPELEC-RAMAN instrument (Metrohm-DropSens), which includes a 785 or 638 nm laser source. More details are in the Supporting Information.

### UV–Vis SEC

*In situ* time-resolved UV–vis SEC was performed using a customized SPELEC instrument (Metrohm-DropSens), which includes a lamp with a halogen and deuterium light source. More details are in the Supporting Information.

### Photoluminescence SEC

*In situ* photoluminescence SEC was performed by using a customized SPELEC instrument (Metrohm-DropSens), using an external LED as a light source (LED-VIS-Kit, Ocean Insight). More details are in the Supporting Information.

### SEC Measurements

All the SEC instrumentations were controlled by DropView SPELEC software (v3.4 20CZ10, Metrohm-DropSens). Detailed information about the setup to perform SEC measurements can be found in the Supporting Information.

### Scanning Electron Microscopy (SEM)

The morphology of the SOERS substrates was studied by using an ultrahigh-resolution field-emission scanning electron microscope, model GeminiSEM560 (Zeiss), applying an electron beam of 2.00 kV and collecting the response of the secondary electrons with an InLens detector.

### X-ray Diffraction (XRD) Measurements

XRD measurements were performed using a D8 Discover Davinci (Bruker) X-ray diffractometer, using a Cu K_α_ radiation source (λ = 0.154 nm). Scans were recorded in the range of 2θ = 5–70°, using a step size of 0.05° and integration of 1 s per step.

### SOERS Substrates

Different SOERS substrates were used throughout this work to demonstrate and study this phenomenon. Experimental details about the preparation of these substrates can be found in the Supporting Information.

## Results and Discussion

### Influence of the Metal Substrate and the Electrolytic Conditions

EC-SOERS has only been reported under specific electrolytic conditions, for silver electrodes and using chloride or bromide as a precipitating agent.^20,23−25,27,38^ This work demonstrates that EC-SOERS can be obtained under a wide range of experimental conditions. When the electrochemical oxidation of a silver electrode is carried out in the presence of species such as KSCN or K_4_Fe(CN)_6_, the Raman enhancement of several molecules is also observed during the release of silver cations, similar to the experiment shown in Figure 1. Figure S1 shows the EC-SOERS signal of a variety of molecules, such as alizarin, caffeic acid, and isonicotinic acid, using different precipitating agents. Likewise, the use of copper electrodes as EC-SOERS substrates, using KI and KSCN as precipitating agents, is also shown (Figure S1e,f), being the first time that Cu electrodes are used as EC-SOERS substrates. In fact, EC-SOERS on Cu surfaces is completely similar to the phenomenon described for Ag. This means that the use of a precipitating agent and the release of copper cations remain mandatory to achieve the enhancement of the Raman signal.

**Figure 2:**
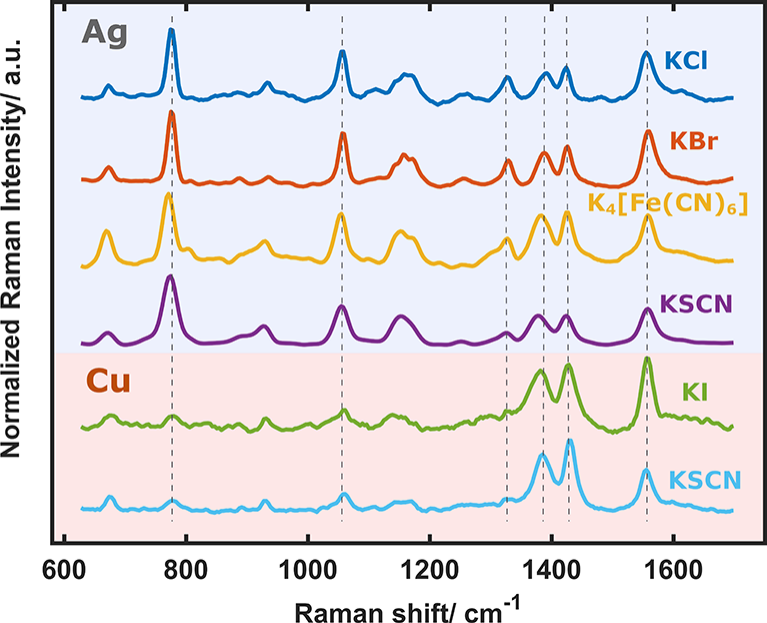
Normalized EC-SOERS spectra of clopyralid using different precipitating agents on different substrates: silver and copper. Experimental conditions are described in Figure S1 in the Supporting Information.

The existence of a variety of EC-SOERS substrates opens new possibilities for analysis and eases the study of the origin of EC-SOERS. Figure 2 shows a comparison of the EC-SOERS spectra for the same molecule on different substrates. A common herbicide, clopyralid, was chosen for this study owing to its difficulty to be detected using SERS but easily detected using EC-SOERS.^23^ As can be observed, the spectra on silver (blue region) are quite similar for the different precipitating agents, whereas the spectra on copper (pink region) present some clear differences with respect to those on the silver ones. These differences, however, only affect the relative intensity of the Raman bands since the Raman shifts of the bands remain constant over all studied substrates. This behavior suggests that the interaction between the target molecule and the substrate is similar for the salts of the same metal but different among the metal substrates, Ag and Cu. This different Raman behavior opens very interesting possibilities for the detection of molecules on a wide range of substrates.

These results demonstrate that EC-SOERS can be produced with different types of crystals, not only for silver salts but also for the copper ones. Furthermore, it can be used for the detection of a wide variety of molecules at low concentrations, which is interesting for the development of new strategies for chemical analysis and sensing.

### Characterization of EC-SOERS Substrates

The nanocrystals generated on the electrode surface during EC-SOERS experiments seem to play a key role for the Raman enhancement since the use of precipitating agents is mandatory to observe EC-SOERS behavior.^20,38^ Therefore, the characterization of these substrates was performed to shed more light on the origin of this phenomenon.

**Figure 3:**
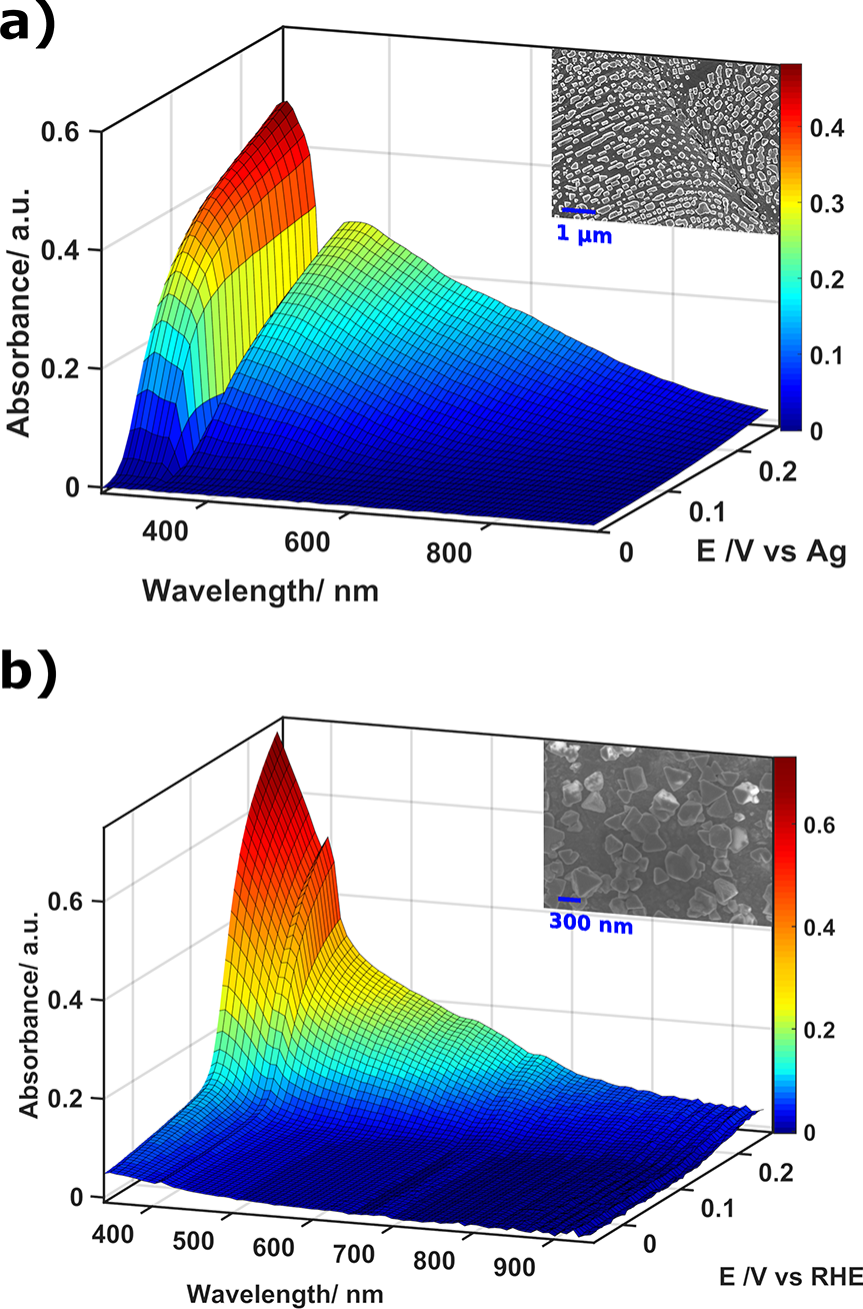
Surface of the UV–vis spectra, obtained by UV–vis absorption SEC in reflection configuration, during the electrochemical generation of two different SOERS substrates: (a) Ag/AgCl and (b) Cu/CuI. Insets show the SEM images of the surface after the experiment. The experimental conditions for the electrochemical generation of these SOERS substrates are summarized in the Supporting Information.

SEM images provide a general view of these substrates (Figure S2 and insets in Figure 3). As can be seen, although only a single anodic linear sweep voltammetry (LSV) is applied to the electrode (Figure S1), a deep change of the surface morphology is generated, yielding nanometer structures on the electrode surface (Figure S2a–f). This is demonstrated by the comparison between the modified and pristine electrode surface (Figure S2g,h). In all cases, nanocrystals of silver or copper salts are generated on the electrode surface after a simple electrochemical treatment. The experimental conditions for the electrochemical generation of these SOERS substrates are summarized in the Experimental Section of the Supporting Information. The nanocrystals present a wide size distribution (from around 100 to 500 nm) among the different studied salts (AgCl, AgBr, Ag_4_Fe(CN)_6_, CuI, and CuSCN). However, the size distribution is quite homogeneous within each sample. Raman analysis (Figure S3a) further confirms the composition of these structures, being notorious for Ag/Ag_4_Fe(CN)_6_, Ag/AgSCN, or Cu/CuSCN due to the characteristic cyanide stretching mode at around 2200 cm^–1^. In addition, Figure S3b shows that there are no differences in the Raman spectra of these substrates when they are generated in the presence of molecules. The study of the Raman spectra of these substrates does not present clear bands between 200 and 800 cm^–1^, corresponding to metallic oxides.

Additionally, *in situ* time-resolved UV–vis absorption SEC in normal arrangement with respect to the electrode surface was performed during the electrogeneration of two different nanocrystals, namely, Ag/AgCl and Cu/CuI, to investigate the optical properties of the generated substrates. Figure 3 shows the growth of defined absorption bands corresponding to AgCl (Figure 3a) and CuI (Figure 3b) crystals during the oxidation of a silver and copper electrode in the presence of the precipitating agent (Cl^–^ for Ag and I^–^ for Cu). The UV–vis spectra agree with those for AgCl and CuI described in literature.^39,40^ However, well-defined absorption bands around 430 nm for AgCl and 580 nm for CuI are observed in our experiments. The growth of these bands completely agrees with the electrochemical generation of these crystals (Figure S4). According to the literature,^29,39,41,42^ these bands could be associated with the presence of defects in the crystal lattice. A similar absorption band can also be observed during the galvanostatic generation of Ag_4_[Fe(CN)_6_] on silver electrodes (Figure S5).

XRD analysis confirms the composition and crystal structure of these substrates in Figure S6. All the XRD patterns match perfectly with the standard data of Ag and AgCl (Figure S6a) and Cu and CuI (Figure S6b).

Although the presence of nanocrystals on the electrode surface is mandatory to observe EC-SOERS, it is not enough to explain the phenomenon since the Raman signal does not increase when the target molecule is added on the electrochemically generated substrate (Figure S7b,c, first 20 s). In fact, previous works describe that when the anodic potential is no longer applied to the electrode, the SOERS effect disappears.^20^ Therefore, the applied potential seemed to play a key role in signal enhancement.

### Demonstration of the Origin of SOERS

During the past few years, our research group has carried out numerous spectroelectrochemistry studies to reveal the role of the applied potential in the EC-SOERS enhancement. This factor always seemed to play a key role for the Raman enhancement since SOERS could only be observed above a certain threshold potential and under potential control.^20^ From the Raman spectral analysis, we proposed that an oxidative deprotonation of the target molecule should take place since the Raman signals corresponding to the deprotonated form of the molecules were always observed.^24,27^ This explanation was previously proposed based on the adsorption of molecules on the crystals of the salt, which seemed to be favored by the potential.^20,38^ However, the new experimental data provide a better insight into the molecule–substrate interaction and clarify the role of the applied potential. Based on new observations, Raman signal enhancement can be obtained without potential mediation, simply by adding a mixture of the target molecule and silver cations on a modified silver electrode (Figure S7b)

The addition of the metal cation to the solution leads to a clear enhancement of the Raman signal of the target molecule. Low concentrations of silver do not produce any amplification of the Raman signal, but from concentrations around 1 mM onward, it is possible to obtain the spectra of the target molecule (Figure S8, green region). This enhancement also occurs using a copper electrode modified with CuI crystals. The modified electrode does not yield any Raman enhancement, but the addition of a Cu(II) solution is enough to generate a clear Raman enhancement of the target molecule (Figure S7c).

This Raman enhancement can be quenched by the addition of any precipitating agent to the solution on the substrate containing the target molecule and the metal cation since metal cations precipitate as insoluble salts, causing the Raman enhancement to vanish abruptly (Figure S9). These experiments demonstrate the mandatory presence of these metal cations to obtain the enhancement of the Raman signal. The generation of the SOERS effect by addition of the corresponding metal cation also leads to some differences in the relative intensity of the Raman spectra of molecules. For example, in the case of fluorescein, the Raman spectrum features depend on the substrate used: Ag/AgCl or Cu/CuI (Figure S10). The fluorescein Raman assignment can be found in Table S1 in the Supporting Information. It is worth noting that no enhancement of the Raman signal is observed when a mixture of target molecules and metal cations is added on the bare metal surface (Figure S8, pink region). This fact reveals that the presence of nanocrystals is mandatory to obtain the SOERS signal. In light of these results, we concluded that the applied potential in EC-SOERS experiments plays two different roles: (i) the electrogeneration of nanocrystals on the electrode surface and (ii) the generation of metal cations in the further anodic region. Thus, the key factor in the amplification mechanism is not the potential but the presence of the metal cation in contact with the nanocrystals on the metal substrates.

**Figure 4:**
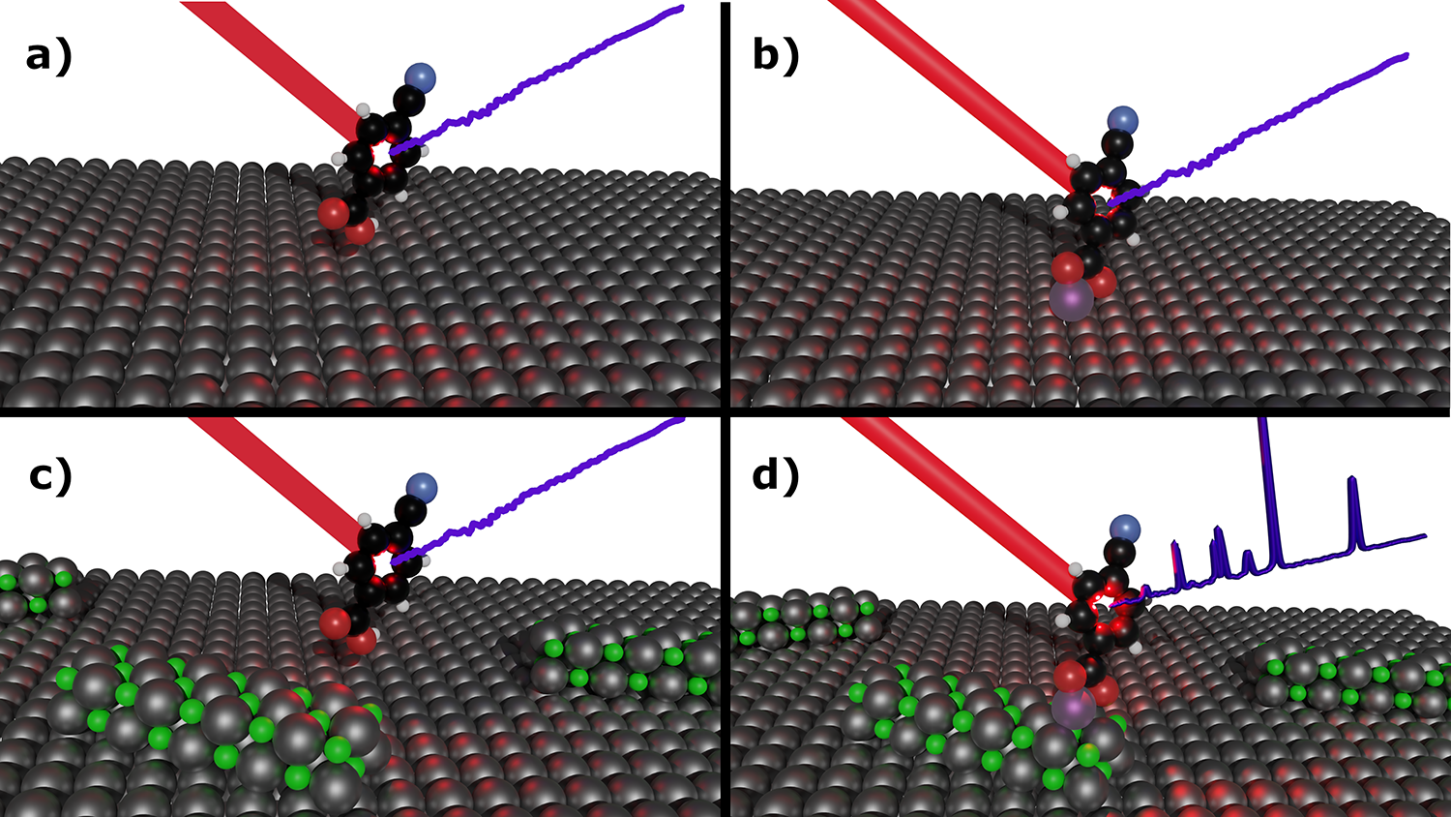
Schematic of the different scenarios that can take place in the SOERS strategy: (a) molecule + metal surface, (b) molecule + metal cation + metal surface, (c) molecule + semiconductor/dielectric crystals + metal surface, and (d) molecule + metal cation + semiconductor/dielectric crystals + surface. It should be noted that only in the last picture (d), a SOERS spectrum is obtained.

Therefore, EC-SOERS should be considered a strategy or methodology to enhance the Raman signal of molecules. Figure 4 shows a schematic summary of the different situations that can take place during the SOERS methodology. First, the molecule can be on the bare metal substrate (Figure 4a), which gives no Raman response, and the presence of metal cations (Figure 4b) is not enough to obtain the Raman enhancement, nor even the presence of the semiconductor/dielectric crystals by themselves gives a SOERS response (Figure 4c). However, it is the interaction between all these elements, metal substrate, semiconductor/dielectric crystals, metal cations, and molecule (Figure 4d), that leads to the Raman amplification by the SOERS strategy.

The fact that the Raman spectra of the molecules are usually those of the deprotonated target molecules^24,27^ led us to think that during the SOERS strategy, some kind of complex is formed between the molecules and the silver or copper cations. This was ruled out in the initial studies of EC-SOERS since no changes were observed in the UV–vis absorption spectra of the molecules when the silver cation was added to a solution containing the target molecule.^20^ Nevertheless, it is possible that surface complexes could play a role in the observed Raman enhancement. It is well known that silver cations can adsorb on silver halide, and therefore, it is possible that these adsorbed silver cations facilitate the generation of these complexes. Moreover, the formation of these complexes can be explained in the terms described by Kolthoff et al. for the titration of chloride with silver cations and detection with fluorescein, known as the Fajans method.^43^ In such an explanation, initially, silver chloride is generated by precipitation, and when a slight excess of silver cation is reached, a fluorescein silver complex is formed on the silver chloride surface, which is noticeable by the color change of the precipitate.

The process that takes place in SOERS seems to be analogous to the phenomenon observed during the titration of chloride with silver. To demonstrate this fact, two different types of *operando* SEC experiments were performed for both silver and copper electrodes.

**Figure 5:**
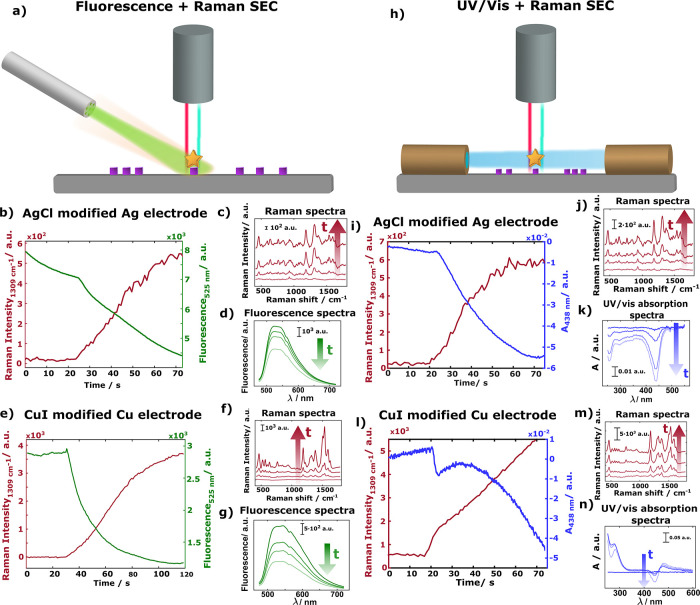
Schematic of the setup to perform simultaneously (a) Raman and fluorescence SEC or (h) Raman and UV–vis SEC in a parallel configuration. Comparison of the evolution of the fluorescence and Raman spectra of fluorescein during the galvanostatic oxidation of (b) a Ag/AgCl electrode or (e) Cu/CuI electrode. Details of the Raman and fluorescence spectra during the experiment are shown in panels (c, d) and (f, g) for Ag and Cu, respectively. Comparison of the evolution of the UV–vis absorption and Raman spectra of fluorescein during the galvanostatic oxidation of a Ag/AgCl electrode (i) or Cu/CuI electrode (l). Details of the evolution of Raman and UV–vis absorption spectra are found in panels (j, k) for Ag and in panels (m, n) for Cu. Experimental conditions are described in Figures S11 and S12 in the Supporting Information.

The first one (Figure 5a–g) corresponds to the combination in a single experiment of Raman and fluorescence SEC, in a galvanostatic experiment, to compare the fluorescence evolution of fluorescein with the corresponding EC-SOERS response. A schematic of the setup used to perform simultaneous fluorescence and Raman SEC is shown in Figure 5a, and a detailed description of this experiment is presented in Figure S11. The second one (Figure 5h–n) is related to the combination, in a single experiment, of UV–vis absorption in a parallel configuration and Raman SEC, to study the electrode/solution interface. The schematic of the setup used to perform simultaneously UV–vis in parallel configuration and Raman SEC is shown in Figure 5h, and a more detailed description is shown in Figure S12.

SOERS substrates were generated by the electrochemical oxidation of the electrode in the presence of the precipitating agent (5 mM KCl/KI + 0.1 M HClO_4_) to generate the corresponding insoluble crystals of AgCl and CuI (for more details, see the Experimental Section in the Supporting Information). Then, the substrate was thoroughly rinsed, and the oxidation of the metal was performed by applying positive currents of 150 μA for Ag and 400 μA for Cu in 0.1 M HClO_4_ + 150 and 100 μM fluorescein for Ag and Cu, respectively. An increase in the Raman signal is obtained concomitantly with the decrease in the fluorescence signal using the two metals due to the formation of the fluorescein silver (Figure 5b–d) and copper (Figure 5e–g) complexes, which is observed in the change of the fluorescence spectra. The decrease in fluorescence is observed when the same experiment is carried out on a nonmodified silver electrode (Figure S13); however, the Raman enhancement is not observed, indicating that a surface process related to the interaction of molecules with metal cations adsorbed on the nanocrystals is taking place when salt nanocrystals are present on the electrode, which is observed in the Raman signal.

On the other hand, when the UV–vis absorption in parallel configuration is registered simultaneously with the Raman response, the evolution of a negative band at 438 nm can be seen, related to the depletion of the fluorescein in the adjacent solution to the electrode due to the adsorption of these molecules on the substrate to generate the fluorescein silver (Figure 5i–k) or copper (Figure 5l–n) complexes. This band is also perfectly correlated with the Raman signal increase, confirming our hypothesis of generation of a metal cation–molecule complex, which is observed in the evolution of the UV–vis spectra, adsorbed on the nanocrystal surface, which is observed in the evolution of the Raman spectra, being responsible for the enhancement of the Raman signal. The two spectroscopic techniques must be performed simultaneously to demonstrate the generation of the metal cation–molecule complex and the concomitant adsorption on the nanocrystal surface.

The number of molecules adsorbed on the surface cannot be obtained only from the fluorescence or UV–vis spectra since it can only be concluded from these optical responses that fluorescein has been chemically modified in solution. These optical signals are proportional to the concentration of the metal cation–molecule complex in the diffusion layer. However, only the Raman response is sensitive to the adsorption of the metal cation–molecule complex. Figure S14 shows the linear correlation between fluorescence at 525 nm vs Raman intensity at 1309 cm^–1^ and absorbance at 438 nm vs Raman intensity at 1309 cm^–1^, indicating that the SOERS signal is linear with the concentration of the metal cation–molecule complex at our time scale. The adsorption of the metal cation–molecule complex on the surface is demonstrated when two complementary spectroscopies are concomitantly used.

In addition, this correlation can be further confirmed with other fluorescent molecules such as riboflavin (Figure S15).

Interestingly, there is a correlation between the signal of the target molecule and the band of the AgCl vibration at 240 cm^–1^ (Figure S16). As can be observed, the trend of the Raman signal corresponding to the target molecule is very similar to the band of the Ag–Cl vibration mode when the silver cation is generated. This correlation further demonstrates that the substrate is activated by the generation of silver cations.

A final experiment that demonstrates the correlation of the amplification of the Raman signal with the concentration of the metal cation formed during the oxidation of the electrode was performed. In this case, the absorbance in a parallel configuration was simultaneously measured during a Raman SEC experiment. As can be seen in Figure S17, the absorbance band at 215 nm, related to free Ag^+^, grows fully correlated with the Raman signal of phthalic acid during the oxidation of a silver electrode in which AgCl was previously deposited using LSV in 5 mM KCl + 0.1 M HClO_4_. A Raman spectrum characteristic of phthalate due to complexation with the silver cations adsorbed on the SOERS substrate is observed.

The contribution of adsorbed species and surface complexes to SERS enhancement on metal nanoparticles has been widely explored since the early years of the field, studying species like chloride, bromide, SCN^–^, or CN^–^.^16,17,44,45^ The adsorption of charged species on SERS substrates has been widely reported to increase the sensitivity of SERS on metal nanoparticles, being an indispensable tool to achieve single-molecule detection.^15^ Studies on this topic revealed that adsorption of ions could induce a secondary adsorption of several analytes, allowing the SERS detection of target molecules.^18,19,46,47^ Most of the reported works use an adsorption strategy to improve the SERS activity of metal nanoparticles. In a similar fashion, the SOERS strategy is based on the use of cations to facilitate the adsorption of molecules on a new family of Raman-enhancing substrates. These materials have not been previously reported in the literature as Raman-enhancing substrates. The synthesis of SOERS substrates can be easily carried out, in a reproducible way, by electrochemical oxidation of the metal in the presence of a precipitating agent. Moreover, SOERS substrates exhibit a very good sensitivity, detecting very different molecules at the nM level (Figure S18).

The set of SEC experiments shown in this work demonstrates that SOERS is a new strategy that amplifies the Raman signal, being controlled by two main factors: (i) the generation of characteristic nanocrystals on a metal surface and (ii) the interaction of molecules with metal cations adsorbed on the nanocrystals. Both nanocrystals and adsorbates are responsible for the amplification of the Raman signal, which may explain the characteristics of the Raman spectra (e.g., the presence of deprotonated species at low pH).

## Conclusions

A new family of Raman-enhancing substrates has been described. EC-SOERS has been reported under very different and novel experimental conditions, including both silver and copper substrates, providing a very valuable tool to detect molecules with diverse chemical structures and obtaining, in a simple experiment, all of the advantages of specificity of Raman spectroscopy together with a high sensitivity and reproducibility.

*Operando* fluorescence and UV–vis SEC experiments demonstrate that the interaction between the molecule and the SOERS substrate is promoted by the adsorbed metal cations on the electrosynthesized crystals, yielding the loss of fluorescence of the probe molecule and the decrease in the concentration observed in parallel UV–vis experiments, showing the potential of SEC to study complex systems.

This work also demonstrates that SOERS is not a potential dependent phenomenon since it can be obtained by generation of nanocrystals on a metal surface and adding the corresponding metal cation to the solution containing the target molecule. However, electrochemistry is still considered the fundamental tool for the generation of active SOERS substrates in a very simple and reproducible way.

The results described in this work demonstrate that the SOERS strategy is related to the generation of metal salt nanocrystals on the metal substrate and the adsorption of the molecules mediated by the adsorbed metal cations. We believe that this strategy could be transferred to other dielectric/semiconductor substrates to promote the detection of target molecules.

These results will be relevant for the design of new SOERS-active substrates that could be of great interest for the study of interfacial processes or for the development of new chemical sensors as interesting as those provided by SERS.

## Supporting Information Available

Experimental section, EC-SOERS response on Ag and Cu substrates, SEM characterization of SOERS substrates, Raman characterization of SOERS substrates, UV–vis characterization of the silver hexacyanoferrate(II) substrate, SOERS generation by addition of metal cations, the effect of the metal cation concentration and chloride addition, EC-SOERS spectra of fluorescein over silver and copper substrates, riboflavin Raman and fluorescence experiment, correlation between photoluminescence/absorbance and Raman signal, correlation of the AgCl Raman band with the fluorescein Raman band, relationship between Ag^+^ generation and Raman enhancement, and improving the sensitivity of EC-SOERS (PDF)
