# Supplementary material for: Raman, UV–Vis Absorption, and Fluorescence Spectroelectrochemistry for Studying the Enhancement of the Raman Scattering Using Nanocrystals Activated by Metal Cations
Source: Anal Chem. 2023 Oct 23;95(44):16070–8. doi: 10.1021/acs.analchem.3c01172 (PMC10633809; doi:10.1021/acs.analchem.3c01172)
Supplement: Supplementary file 1 — ac3c01172_si_001.pdf [file ac3c01172_si_001.pdf]

Supporting information

Raman, UV/Vis absorption and fluorescence spectroelectrochemistry for studying the enhancement of the Raman scattering using nanocrystals activated by metal cations.

Sheila Hernandez[1], Martin Perez-Estebanez[1], William Cheuquepan[1,2,3], Juan V. Perales-Rondon[1], Aranzazu Heras*[1], Alvaro Colina*[1]

[1]Department of Chemistry, Universidad de Burgos, Pza. Misael Bañuelos s/n, E-09001 Burgos, Spain

[2]Bernal Institute, University of Limerick (UL), Limerick, V94 T9PX, Ireland

[3]Department of Chemical Sciences, School of Natural Sciences, University of Limerick (UL), Limerick, V94 T9PX, Ireland

Email: acolina@ubu.es; maheras@ubu.es

Table of content

Detailed experimental section. 3

- Spectroelectrochemistry (SEC) instrumentation. 3
- Spectroelectrochemical measurements 3
- SOERS substrates generation 4

EC-SOERS response on Ag and Cu substrates 5

SEM characterization of SOERS substrates 7

Raman characterization of SOERS substrates 8

Comparison of the LSV and the derivative of the UV/Vis spectra 10

Absorption UV/Vis characterization of silver hexacyanoferrate (II) substrates 11

XRD Analysis 12

Generation of the SOERS effect by addition of metal salts 13

SOERS spectra of fluorescein on silver and copper substrates 17

Detailed description of photoluminescence and Raman SEC experiments 19

Raman and photoluminescence of Fluorescein in absence of SOERS substrate 23
Correlation between photoluminescence/absorbance and Raman signal 24
EC-SOERS response of riboflavin by galvanostatic pulses 25
Correlation of the AgCl Raman band with the fluorescein Raman band. 26
Correlation between EC-SOERS and Ag^{+} concentration 27
Improving the sensitivity of EC-SOERS 28
References 29

Detailed experimental section.

Spectroelectrochemistry (SEC) instrumentation.

Raman SEC. Two different laser wavelengths were used for the *in-situ* Raman SEC experiments (785 nm and 638 nm). The laser power in all experiments was set at 80 mW (254 W cm^{-2}) for the 785 nm laser source and 20 mW (63.72 W cm^{-2}) for the 638 nm laser source. This instrument was connected to a Raman probe (DRP-RAMANPROBE, Metrohm-DropSens). An integration time of 1 s is used in all experiments.

UV/Vis SEC. Two different configurations were used along the UV/Vis SEC experiments: normal and parallel configuration. In normal arrangement, the incident light beam goes perpendicular to the surface providing information on both the diffusion layer species and the electrode surface. In parallel arrangement, the light beam passes parallel to the electrode surface, providing information only from the first 100-120 µm of solution surrounding the electrode[1].

The instrument was connected to a reflection probe (DRP-RPROBE, Metrohm-DropSens) to perform experiments in normal configuration, or two bare optical fibers (100 µm, Avantes) fixed and aligned parallel to the electrode to measure in parallel configuration.

Photoluminescence SEC. *In-situ* photoluminescence SEC was performed using a customized SPELEC instrument (Metrohm-DropSens), using an external LED as a light source (LED-VIS-Kit, Ocean Insight). The LED source was connected to a reflection probe (200 µm, Avantes). We used two different LEDs as excitation source: 405 nm for fluorescein and 385 nm for riboflavin. Power of the LED source was adjusted for each experiment to not overexpose the detector, with an integration time of 100 ms.

Spectroelectrochemical measurements

Two different electrochemical cells were used to perform the various spectroelectrochemical measurements. For most cases, a home-made cell for screen-printed electrodes (SPEs) were used. The SPEs (AgSPEC013, AgSPE010, Cu10SPE, Metrohm-DropSens) consist of a working electrode of 4 mm diameter (1.6 mm for AgSPEC013) made with silver or copper ink, a counter electrode of carbon ink and a pseudo-reference electrode of silver ink. A volume of 50 µL of solution placed on the three electrodes system was used when working with SPEs.

Although CuSPE were used for some experiments, the quality of the copper ink is not optimal for performing EC-SOERS experiments. Thus, for most experiments involving copper substrates, a different electrochemical cell was used: a small Teflon cell with a capacity of 3 mL was used to hold a classical 3 electrode system, using a copper rod (3.18 mm diameter, Alfa Aesar, >99.999 %) as working electrode, a gold foil (60x3 mm) as counter electrode and a reversible hydrogen electrode as reference. Before each experiment, the copper working electrode were polished until mirror-like finish and then electropolished in a mixture of H_3PO_4:H_2SO_4:H_2O 10:5:2 at +3 V vs Cu for 10 seconds two times, rinsing the electrode with pure water between electropolishing cycles.

When several spectroelectrochemical techniques were performed simultaneously, e.g., Raman and photoluminescence SEC, Raman reflection probe were focused on the electrode surface in normal configuration (perpendicular to the electrode). The second reflection probe, which provides the UV/Vis or photoluminescence information, were focused on the electrode surface in diffuse reflectance configuration (incidence angle <45º). This probe was located close to the electrode surface, to minimize the contribution of the bulk solution to the spectroscopic signal.

SOERS substrates generation

In addition to the classical silver EC-SOERS substrates described in Figure 1 and Figure S1, different SOERS substrates are used throughout this work to demonstrate this phenomenon. These substrates are generated electrochemically, and the procedure can be summarized as follows:

Ag/AgCl SOERS substrate. A LSV is carried out between 0.00 V and +0.25 V vs Ag at 0.02 V s^{-1} using AgSPEs and 5 mM KCl and 0.1 M $HClO_4$ as supporting electrolyte.

Ag/AgBr SOERS substrate. A LSV is carried out between 0.00 V and +0.25 V vs Ag at 0.02 V s^{-1} using AgSPEs and 5 mM KBr and 0.1 M $HClO_4$ as supporting electrolyte.

Ag/$Ag_4[Fe(CN)_6]$ SOERS substrate. A LSV is carried out between -0.10 V and +0.25 V vs Ag at 0.02 V s^{-1} using AgSPEs and 1 mM $K_4[Fe(CN)_6]$ and 0.1 M $HClO_4$ as supporting electrolyte.

Ag/AgSCN SOERS substrate. A LSV is carried out between -0.05 V and +0.25 V vs Ag at 0.02 V s^{-1} using AgSPEs and 1 mM KSCN and 0.1 M $HClO_4$ as supporting electrolyte.

Cu/CuI SOERS substrate. A LSV is carried out between -0.10 V and +0.25 V vs RHE at 0.02 V s^{-1} using Cu rod electrode and 5 mM KI and 0.1 M $HClO_4$ as supporting electrolyte.

Cu/CuSCN SOERS substrate. A LSV is carried out between +0.10 V and +0.30 V vs RHE at 0.02 Vs^{-1} Cu rod electrode and 1 mM KSCN and 0.1 M $HClO_4$ as supporting electrolyte.

EC-SOERS response on Ag and Cu substrates

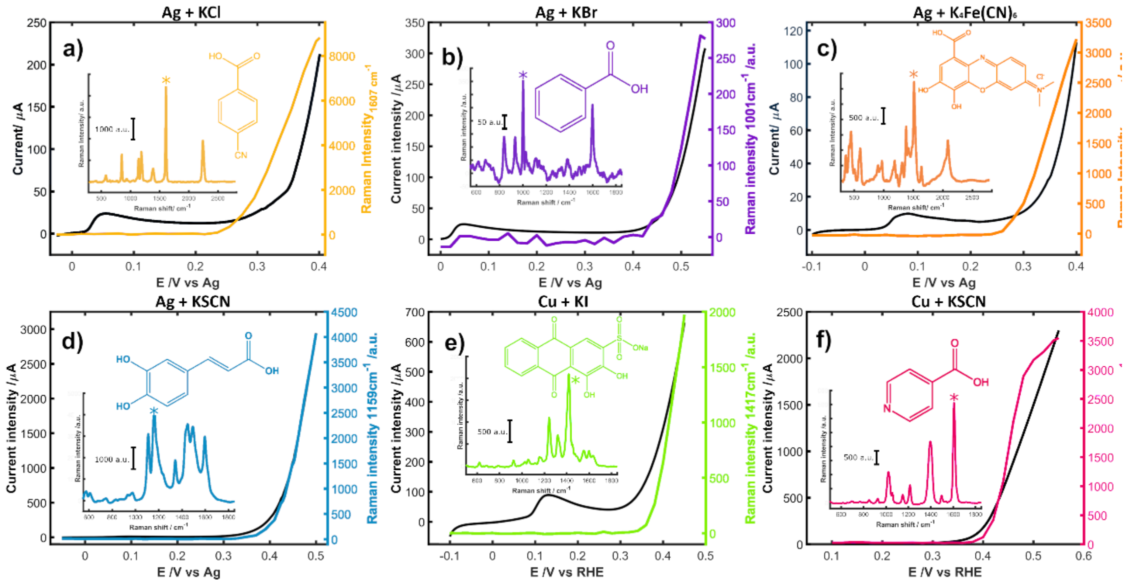

*Figure S1. LSV and EC-SOERS responses of several analytes in different electrolytic media. (a) 0.2 mM 4-cyanobenzoic acid + 5 mM KCl + 0.1 M $HClO_4$, LSV from -0.025 V to +0.40 V (b) 2.5 mM benzoic acid + 5 mM KBr + 0.1 M $HClO_4$, LSV from 0.00 V to +0.55 V (c) 0.4 µM gallocyanine + 1 mM $K_4Fe(CN)_6$ + 0.1 M $HClO_4$, LSV from -0.10 V to +0.40 V (d) 25 µM caffeic acid + 1 mM KSCN + 0.1 M $HClO_4$, LSV from -0.05 V to +0.50 V (e) 10µM alizarin RS + 5 mM KI + 0.1 M $HClO_4$, LSV from -0.10 V to +0.45 V (f) 0.1 mM isonicotinic acid + 1 mM KSCN + 0.1 M $HClO_4$, LSV from +0.10 V to +0.50 V. The scan rate of all experiments is 0.02 $V{\cdot}s^{-1}$. Experiments a-d were performed on AgSPEs (a-c, AgSPE C013, d, AgSPE010), using a 785 nm laser source. Experiments e-f were performed on Cu electrodes, using a 638 nm laser source. For each molecule, the intensity of the main Raman band (labelled with *) is plotted versus the applied potential in the right axis. In each case inset shows a Raman spectrum of the molecule at the potential for the maximum enhancement of the Raman signal, and the molecular structure of the target molecule.*

Figure S1 shows the EC-SOERS response of a variety of molecules on Ag and Cu electrodes, using different electrolytic conditions and metal substrates to enhance its Raman response. EC-SOERS can be observed both on silver (previously reported) and copper electrodes, which is shown here for the first time. The different precipitating agents give rise to different metal salt crystals on the electrode surface. In most cases, the generation of those crystals is marked in the LSV by an anodic process around ~ +0.1 V. For the KSCN, however, the formation of AgSCN or CuSCN is not easily observed in the LSV because of the low voltammetric signal associated with this process. However, it is well described in the literature the formation of these insoluble species in the presence of KSCN.[2–6]

From the figure it can be inferred that several metal salts enable the enhancement of the Raman signal of molecules, including AgCl, AgBr, $Ag_4[Fe(CN)_6]$, AgSCN, CuI and CuSCN. For most cases, Raman signal of the molecule increases simultaneously with the anodic current corresponding to the massive dissolution of the electrode surface. For certain cases, like 4-cyanobenzoic acid (Figure S1A) or gallocyanine (Figure S1C), the Raman enhancement begins at lower potentials, around +0.25 V, where small currents associated with the generation of Ag^+ are observed. This figure summarizes that EC-SOERS effect can be observed on a variety of substrates, and can be used to enhance the Raman signal of different target molecules, being especially sensitive for molecules containing carboxyl and carbonyl groups, although it can be used to detect analytes with different functional groups, such as sulfonate, as is the case of Alizarin RS (Figure S1E), among others.

It must be noted that, for the studied analytes, EC-SOERS behavior of a certain analyte behavior can be observed on multiple substrates. E.g., Isonicotinic acid (Figure S1F) exhibits EC-SOERS enhancement not only on copper substrates with KSCN, but also over all the electrolytic media shown in Figure S1 (data not shown).

SEM characterization of SOERS substrates

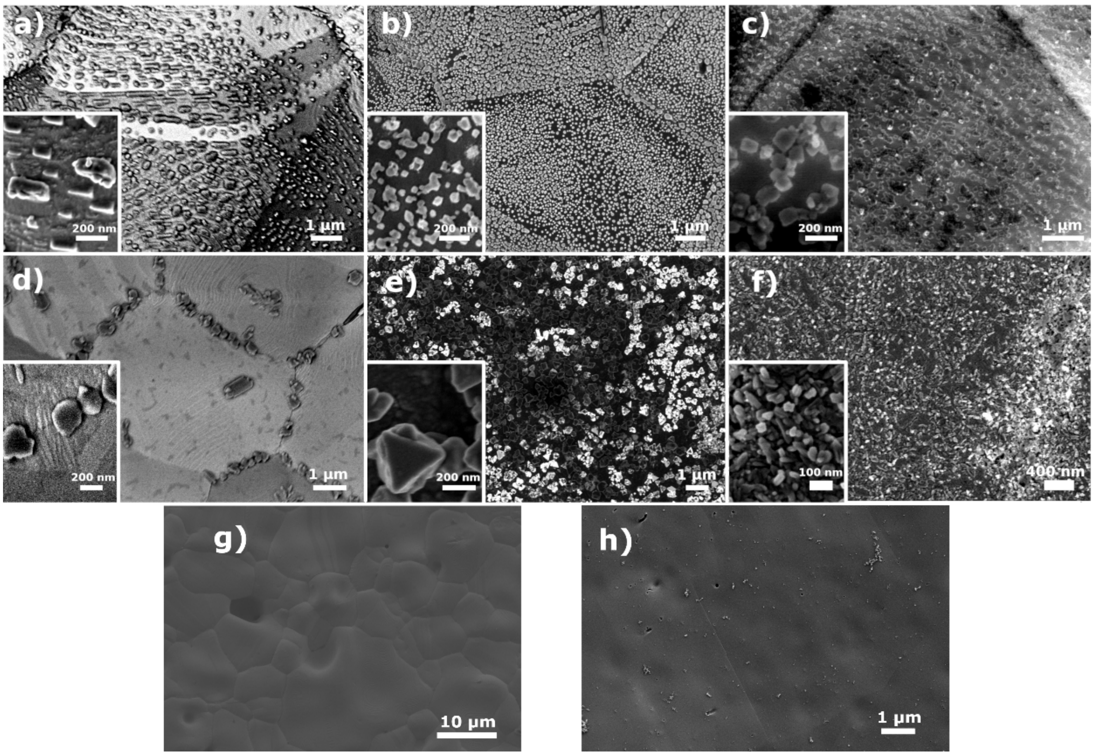

Figure S2. SEM images of the electrogenerated SOERS substrates: (a) Ag/AgCl, (b) Ag/AgBr, (c) $Ag/Ag_4[Fe(CN)_6]$ (d) Ag/AgSCN, (e) Cu/CuI, (f) Cu/CuSCN. All substrates have been generated following the electrochemical protocols described in the experimental section. Insets shows a higher magnification in a certain region of the SEM image. SEM images of pristine electrodes without electrochemical modification are shown in (g) Ag electrode and (h) Cu electrode.

Figure S2 shows the SEM images of different electrogenerated substrates. Different shapes (i.e. rectangular for Ag/AgCl and triangular for Cu/CuI) and sizes (from around 30-40 nm the smallest crystals in $Ag/Ag_4[Fe(CN)_6]$ or Cu/CuSCN to 200-250 nm for Ag/AgCl or Cu/CuI) of the metal salt crystals can be observed, as well as the coated surface, i.e. showing a high coating on the Ag/AgBr or with a low coating on the Ag/AgSCN at which crystals are only observed at the edges of the facets of the electrode. The insets show a higher magnification SEM image to better appreciate the details. Inset in Figure S2a shows how the crystals (Ag/AgCl) emerge from the surface.

SOERS substrates synthesis was carried out as described above. After the electrochemical modification, the substrates were rinsed with water and dried under a nitrogen flow.

Pristine electrodes without electrochemical modification are shown in Figure S2g and h. The comparison between pristine electrodes and SOERS substrates (Figure S2a-f) clearly demonstrates the effect of the electrochemical generation of nanostructures on the electrode surface.

Raman characterization of SOERS substrates

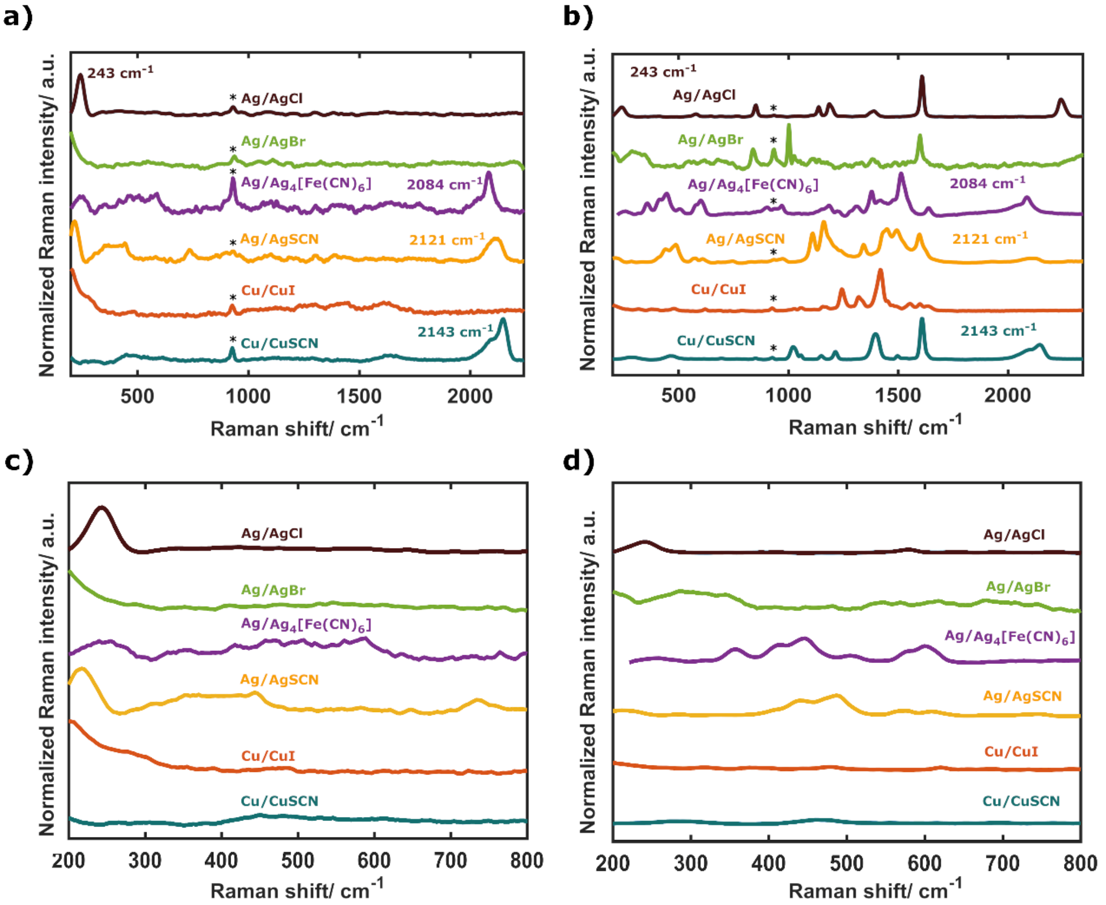

*Figure S3. Normalized Raman spectra of the generated SOERS substrates: Ag/AgCl, Ag/AgBr, Ag/$Ag_4[Fe(CN)_6]$, Ag/AgSCN, Cu/CuI, Cu/CuSCN in the absence (a) and in the presence of a test molecule (b). A magnification of the 200-800 cm^{-1} is presented in the (c) absence and (d) presence of target molecule. All substrates have been generated following the electrochemical protocols described in the experimental section. Raman bands labelled with * are related to the perchlorate ion used as supporting electrolyte.*

Figure S3a shows the Raman spectra of the different electrogenerated SOERS substrates. It should be noted that all the substrates are generated in 0.1 M $HClO_4$, which explains the Raman band at 934 cm^{-1} related to perchlorate ion vibration. The Ag/AgCl substrate spectrum presents a well-defined Raman band at 243 cm^{-1}, which is related to Ag-Cl vibration.[7] For Ag/AgBr substrate, no significant Raman band is observed, which is in agreement with the literature, since the Ag-Br vibration rests on a lower frequency region.[8,9] On the other hand, in the case of Ag/$Ag_4[Fe(CN)_6]$, silver hexacyanoferrate is not widely reported in literature, however, the band at 2084 cm^{-1} may be assigned to the CN A1g vibration.[10,11] Noticeable, this band undergoes a blue shifting compared to the one for potassium hexacyanoferrate at 2094 cm^{-1}, this blue shifting is expected due to the exchange of potassium for silver in the hexacyanoferrate. For Ag/AgSCN spectrum, a broad Raman band at 2121 cm^{-1} is observed, which agrees with that reported in literature for AgSCN vibration.[12] Cu/CuI substrate spectrum is quite similar to Ag/AgBr, without a defined Raman band, this is due to the low frequency vibrations related to CuI.[13,14] Finally, the Cu/CuSCN substrate spectrum presents a well-defined band at 2143 cm^{-1} that can be associated to the CuSCN vibration.[15,16] On the other hand, Figure S3b shows the same substrates when they are generated in presence of a test molecule during a classical EC-SOERS experiment, test molecules and conditions used are the same as Figure S1. As can be observed, there are no differences in the substrate Raman bands when the substrates are generated in presence/absence of target molecules.

All the Raman spectra shown in Figure S3 have been normalized respect to the maximum intensity of each spectrum.

Figures S3c and d provide a magnification of the Raman spectra in the region between 200 and 800 cm^{-1}. The Raman bands in this spectral region confirm that no metallic oxides are observed in the system.

Comparison of the LSV and the derivative of the UV/Vis spectra

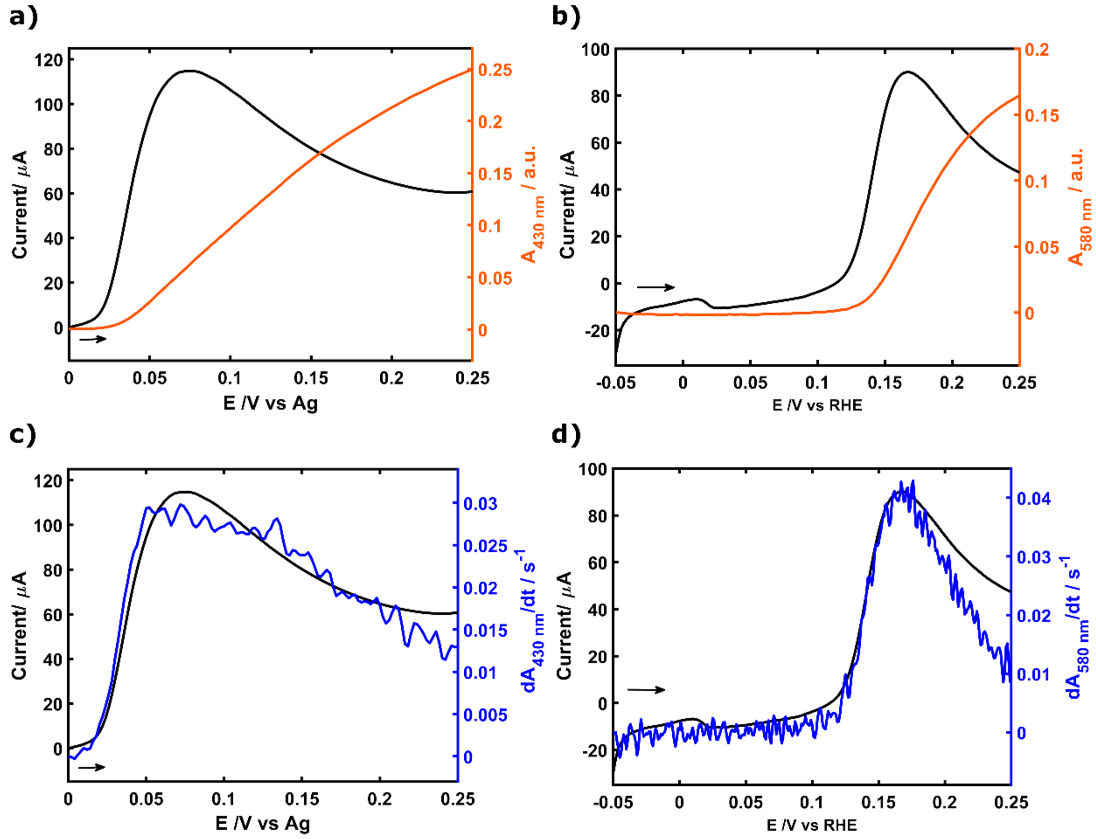

Figure S4. Comparison of LSV (black line) and evolution of the absorbance (orange line) or the derivative of the absorbance (blue line) at 430 nm for AgCl substrate (a and c) and at 580 nm for CuI substrate (b and d).

Figure S4 shows the evolution of the absorption bands at 430 nm for the AgCl substrate and at 580 nm for the CuI substrate, shown in Figures 3a-b. These substrates were electrochemically generated as described in the experimental section of SI. In this case, time-resolved UV/Vis absorption SEC in normal configuration was used to analyze the substrate changes. The evolution of the absorbance versus the applied potential (voltabsorptogram, orange line) at a given wavelength is compared with the LSV (black line) in Figures S4a-b. It can be noted that the absorbance increases concomitantly with the electrochemical process. This match is more clearly seen in Figures S4c-d, where the derivative of the voltabsorptogram (blue line) overlaps with the LSV (black line). From this figure it can be inferred that these absorption bands are unequivocally related to the electrochemical generation of AgCl and CuI.

Absorption UV/Vis characterization of silver hexacyanoferrate (II) substrates

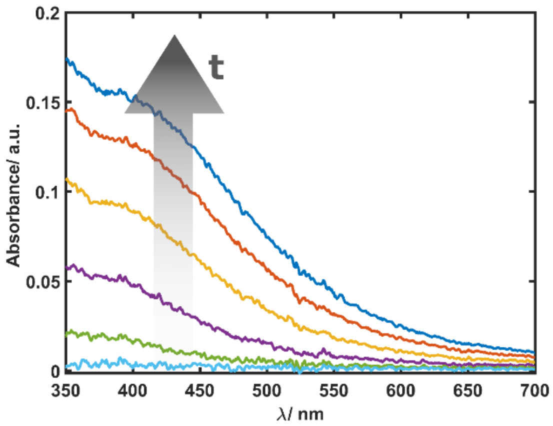

Figure S5. UV/Vis absorption spectra of a AgSPE during its galvanostatic oxidation (10 µA) during 30 s. Electrolytic conditions: 0.1 M $HClO_4$ + 0.1 mM $K_4[Fe(CN)_6]$.

Figure S5 shows the absorption spectra measured in normal configuration, during a galvanostatic oxidation of a AgSPE by applying +10 µA in an electrolytic media containing 0.1 mM $K_4Fe(CN)_6$ and 0.1 M $HClO_4$. Similar to the behavior observed for CuI or AgCl (Figure 3), the formation of silver hexacyanoferrate (II) crystals can be followed by the evolution of a broad absorption band centered at 400 nm.

XRD Analysis

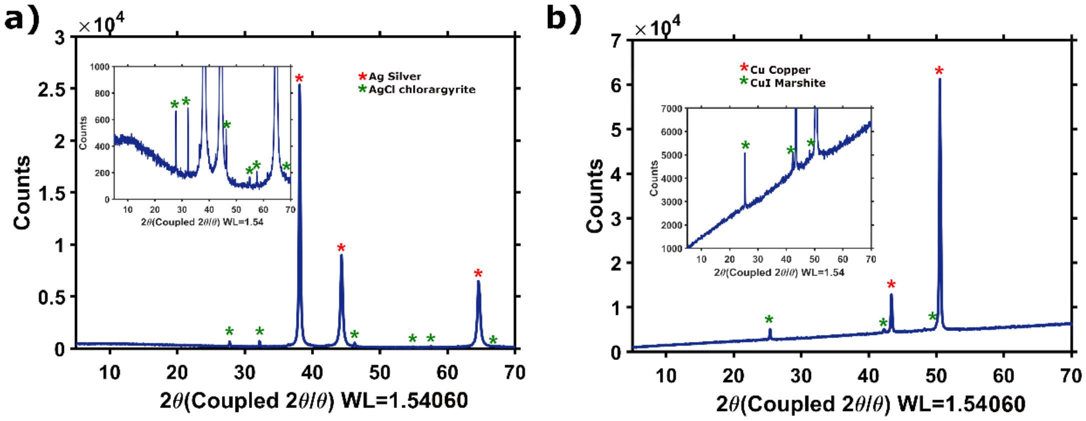

Figure S6. XRD spectra of the Ag/AgCl (a) and Cu/CuI (b) substrates. An amplification of the XRD spectra is shown in the inset in order to better appreciate the peaks related to AgCl and CuI.

Figure S6a shows three intense diffraction peaks (2θ) at 38.1° (111), 44.3° (200) and 64.4° (220), related to Ag metal (ICSD 044387) substrate below the crystals (red *). The other peaks that can be found in Figure S6a (green *) at 27.8° (111), 32.2° (200), 46.2° (220), 54.8° (311), 57.5° (222) and 67.4° (400) are related to the chlorargyrite crystal structure of AgCl particles (ICSD 064734). On the other hand, Figure S6b shows the XRD spectra for Cu/CuI substrates, where the most intense peaks (2θ) at 43.3° (111), 50.4° (200) (red*) are related to the Cu metal (ICSD 064699) substrate below the particles and the other peaks at 25.4° (111), 42.2° (220) and 49.9° (311) (green *) are related to the marshite crystal structure of CuI particles (ICSD 080218).

Generation of the SOERS effect by addition of metal salts

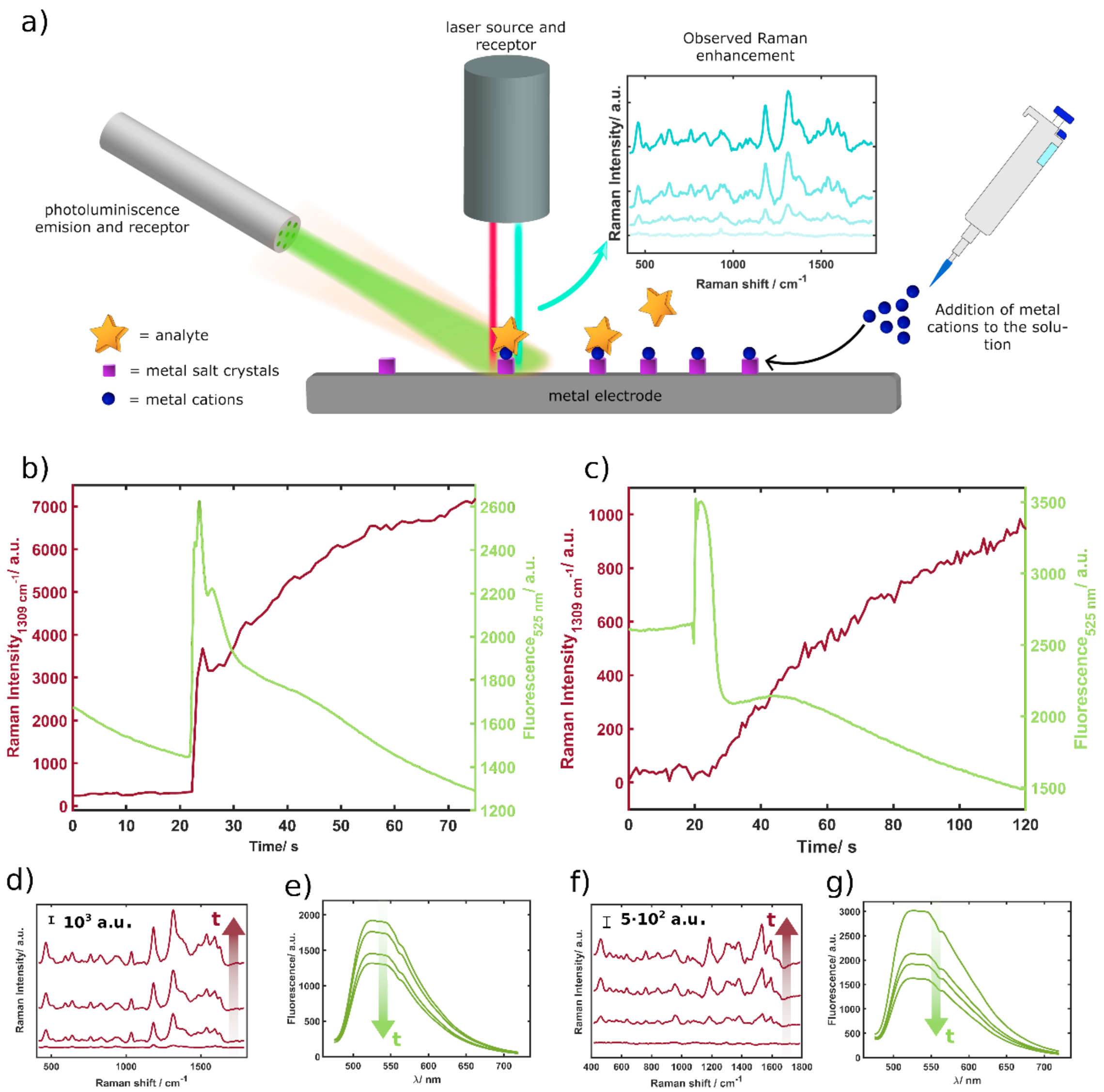

Figure S7. a) Schematic of the experiment. Simultaneous evolution of photoluminescence (green line, right axis) and Raman (garnet line, left axis) of fluorescein on Ag/AgCl (b) or Cu/CuI (c) SOERS substrates. At t=20 s, aliquot of $AgClO_4$ (b) or $CuSO_4$ (c) is added to the system, notably modifying both spectroscopic signals. Details of the Raman and fluorescence spectra are plotted in d-e (fluorescein on Ag/AgCl) and f-g (fluorescein on Cu/CuI).The experimenst were performed at open circuit potential (OCP).

Figure S7 shows the Raman and the fluorescence evolution of fluorescein on two different SOERS substrates: Ag/AgCl and Cu/CuI.

The SOERS substrates were electrochemically generated as described in the experimental section of SI (SOERS substrate generation) without adding fluorescein. In this experiment, CuSPE were used to ease the addition of copper cations, thanks to the smaller volume of solution needed with this electrochemical setup.

Once the SOERS substrates were generated, 50 µL of 150 µM fluorescein for Ag/AgCl SOERS substrate or 50 µl of 100 µM fluorescein for Cu/CuI SOERS substrate were added, both solutions in 0.1 M $HClO_4$. Immediately, the evolution of Raman and fluorescent spectra were registered simultaneously. Initially, in neither of the two experiments shown in Figure S7, the characteristic

Raman spectrum of fluorescein is observed when fluorescein is added on SOERS substrates. However, when 10 μL of a salt of the corresponding metal cation ($AgClO_4$ or $CuSO_4$) is added to the solution, achieving a final concentration of 10 mM Ag^+ or Cu^{2+}, both signals show variations.

The fluorescence spectrum of fluorescein (green lines, Figures S7b and S7c) changes significantly in both substrates due to the perturbation of the added solution on the optical system (from 20 to 25 s). After this perturbation, a general trend in which photoluminescence decreases while the Raman enhancement rises is observed. It should be remarked that the enhancement of the Raman signal of fluorescein is observed instantaneously after the addition of Ag^+ to the system Ag/AgCl SOERS substrate. However, it shows a little delay in the Cu/CuI system.

The evolution of the Raman enhancement can be more irregular than when metal cations are electrochemically generated (Figure 5). This behavior is related to the different local concentrations of metal salts generated when the Ag^+ or Cu^{2+} solution is added, which can generate some irreproducibility. Therefore, although the results shown in this Figure can be considered qualitative experiments, they indicate that in order to observe the EC-SOERS enhancement of fluorescein, the conjugation of three factors is mandatory: the target molecule (fluorescein), the dielectric/semiconductor crystal (AgCl or CuI in this case) and the metal cation (Ag^+/Cu^{2+} in this case).

Effect of metal cations concentration on SOERS enhancement

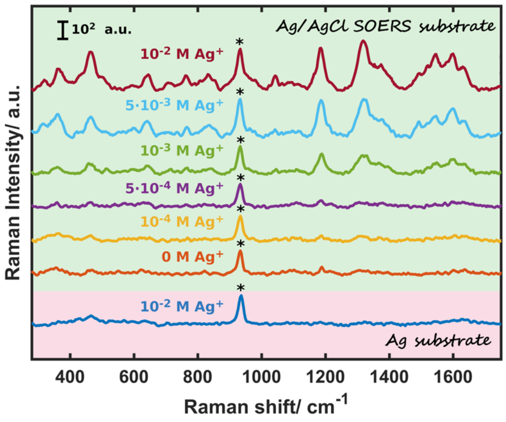

*Figure S8. Raman spectra of 150 µM fluorescein on a bare Ag electrode (bottom, blue spectrum in pink region) and on an electrogenerated Ag/AgCl SOERS substrate (spectra in green region) with addition of different $AgClO_4$ concentrations. All solutions were prepared in 0.1 M $HClO_4$. Raman bands labelled with * are related to the perchlorate ion.*

The Raman enhancement of the molecules is highly dependent of the metal cation concentration (Ag^+ in this figure), as can be seen in green region in Figure S8. Also, the presence of the semiconductor/dielectric nanocrystals is mandatory to obtain the SOERS spectra, since the solution containing the target molecule and a high concentration of Ag^+ on a bare Ag substrate does not provide the characteristic Raman spectrum of fluorescein (pink region).

Effect of chloride addition on SOERS enhancement

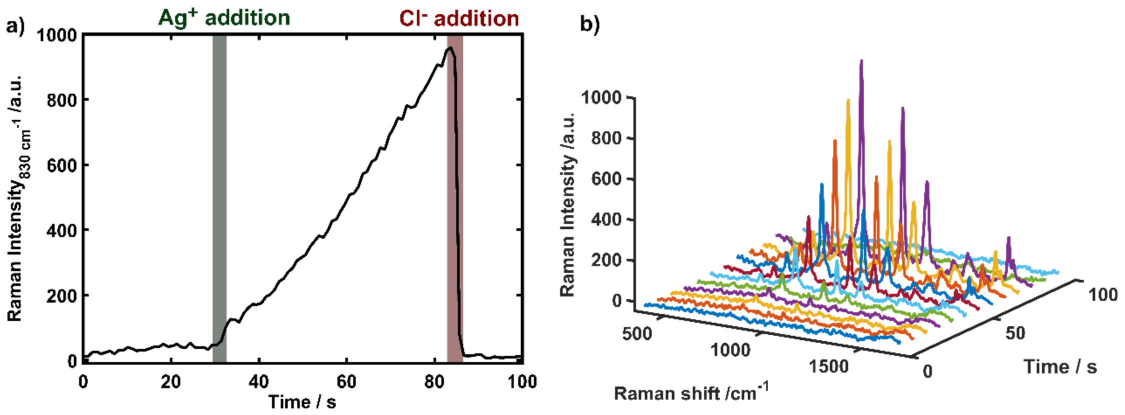

Figure S9. a) Evolution of the characteristc Raman band at 830 cm^{-1} for phathalic acid with time on a Ag/AgCl SOERS substrate. It can be seen the enhancement of the Raman signal when 10 mM Ag^+ is added, around 30 s after starting the experiment, and the vanishment of the spectra when around 5 mM Cl^- is added (≈83 s). b) Raman spectra evolution during this experiment where is displayed one spectrum every 8 s. Ag/AgCl SOERS substrate has been generated following the procedure described in the experimental section of SI.

Figure S9 shows that the presence of Ag^+ cations in solution is necessary to produce the SOERS enhancement. When Ag^+ cations precipitate by addition of Cl^- (or any other precipitant agent), the Raman signal relative to the target molecule (phthalic acid in this case) disappears. This experiment was performed with a Ag/AgCl SOERS substrate electrogenerated as described in the experimental section of SI.

SOERS spectra of fluorescein on silver and copper substrates

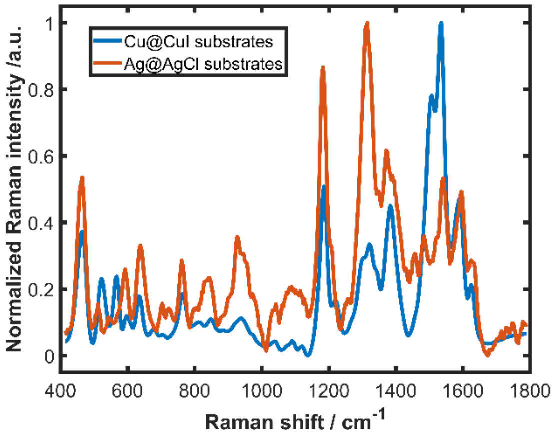

Figure S10. Comparison of the Raman spectra of fluorescein on Ag/AgCl (orange line) and Cu/CuI (blue line) SOERS substrates. The experimental conditions are to the same as those shown in Figure S5.

Table S1. Raman bands assignment of fluorescein, by comparison with literature[17] assignment and the experimental spectra on Ag/AgCl and Cu/CuI SOERS substrates. Raman shifts are given in cm^{-1}.

**dianionic form[18]**	**monoanionic form[18]**	**SOERS spectra on Ag\AgCl substrate**	**SOERS spectra on Cu\CuI substrate**	**Assignment[18]**
-	-	366	359	-
-	-	461	468	-
-	-	-	525	-
-	-	-	571	-
-	-	592	603	-
-	-	637	637	-
-	-	764	770	-
1170	-	-	-	CCH bend (H26, H28, H29, H30)
-	1184	1182	1186	C-OH bend + CCH (H26, 28, 29, 30)
-	-	1206 (sh)	1222 (sh)	-
1311	-	1315	1321	Phenoxide ion stretching conjugated with xanthene ring stretching
1330	1327	1348 (sh)	1341 (sh)	Xanthene ring stretching
-	1414	1371	1385	CCH bend, C-C stretching
1463	1465	1455	-	C-C stretching of xanthene ring + CO stretching OR symmetric COO- stretching
1499	1497	1484	1509	Central ring breathing, C-C stretching (C12-C13 and C5-C6)
1546	-	1540	1535	Xanthene ring C-C stretching
-	1556	-	-	Xanthene ring C-C stretching
1575	-	1580	1580 (sh)	Xanthene ring stretching OR
-	1596	1594	1594	Xanthene ring C-C stretching
1636	1636	1627	1627	Symmetric CO stretching and xanthene ring stretching

Figure S10 shows the SOERS spectra of fluorescein observed in Figure 5, during the galvanostatic oxidation of a Ag/AgCl or Cu/CuI SOERS substrate. When compared, it is clear that the two spectra share the main features, since they are composed by the same number of bands at nearly identical Raman shift. From the assignment table (Table S1), it is observed that the recorded spectra share some features with the reported spectra of the two anionic forms of fluorescein. Wang *et al.* reported that the band at 1184 cm^{-1}, corresponding in part to the phenoxide stretching, can be used as a marker of the protonation state of fluorescein[18], being shifted to higher wavelengths when the molecule is in monoanionic form. In our experiment, a well-defined peak at 1182 cm^{-1} for Ag and the corresponding one at 1186 cm^{-1} for Cu are observed, which denotes

the presence of the monoanionic form. However, some other characteristic peaks of the dianionic specie are present, such as the peak at 1315 cm^{-1}, which is the main peak in the Raman spectra on Cu/CuI substrates. Thus, a mixture of protonation states can be present in the system.

The most noticeable difference between spectra remains in the relative intensity of the bands. The main Raman bands of the spectra are 1580 cm^{-1} for copper substrates and 1315 cm^{-1} for silver substrates, and several other differences in the relative intensity of peaks are observed in the region between 1500–1650 cm^{-1}. This could be attributed to different charge transfer processes or other chemical enhancement related processes. It is well known that the charge transfer processes can enhance selectively some of the Raman bands of the analyte[19–21]. Thus, the intensity changes in the spectra can be related to different charge transfer occurring between the adsorbed cations (Cu^{2+} on CuI or Ag^{+} on AgCl).

Detailed description of photoluminescence and Raman SEC experiments

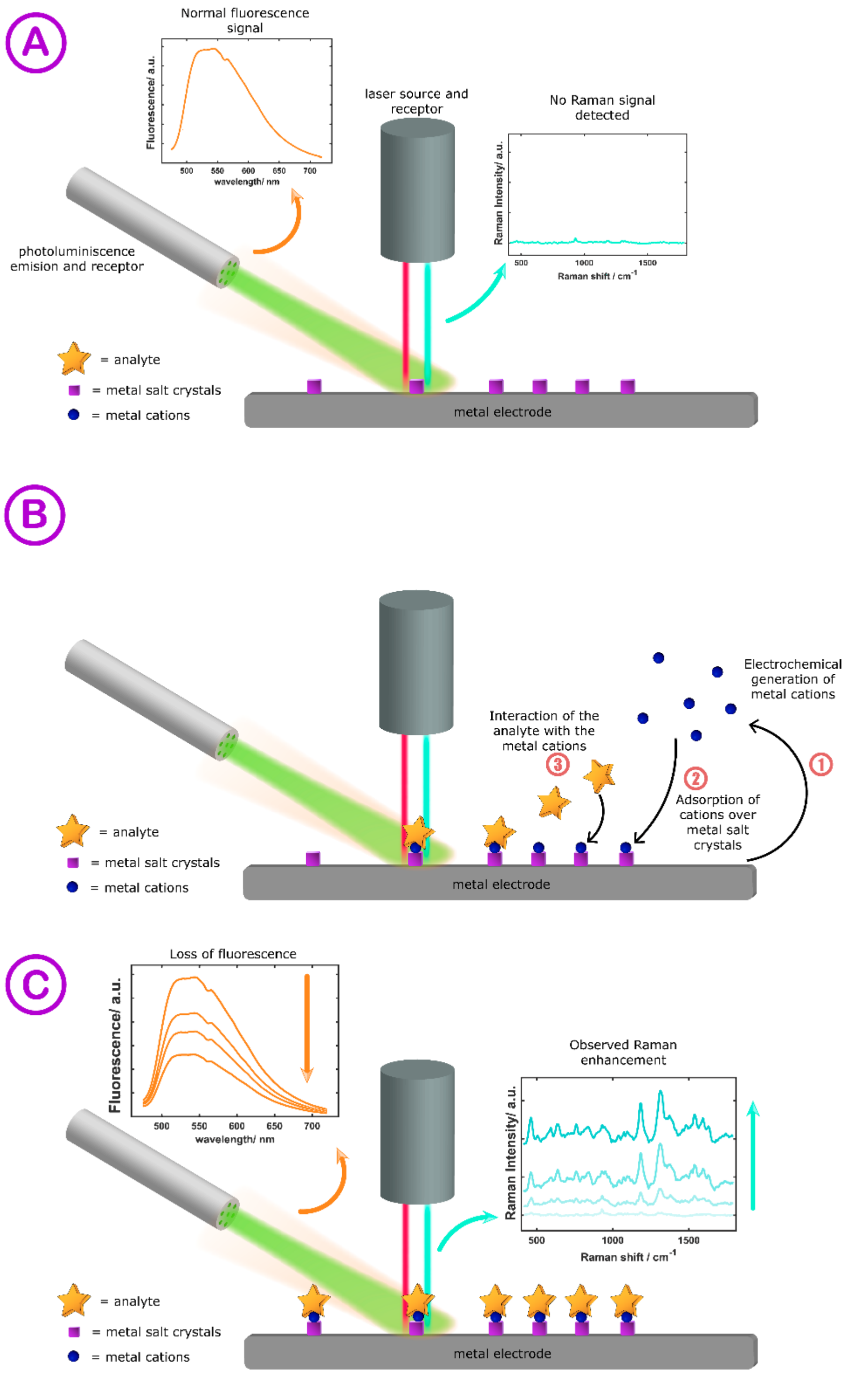

Figure S11. Schematic of the description and interpretation of the experiments shown in Figure 5 (b-g). The schematic shows the proposed evolution of the system during the electrochemical oxidation of a SOERS substrate in this case, Ag/AgCl or Cu/CuI, in presence of a target molecule: (a) Initial system state where no potential is applied to the system; (b) EC-SOERS experiment in which sufficiently anodic potential is applied to partially oxidize the metal surface of the electrode; (c) Interaction between the target molecule and the crystals occurs and changes in both, fluorescence and Raman signals are observed.

Figure S11 shows a schematic of the experimental setup for the acquisition of photoluminescence and Raman signals during the simultaneous SEC experiments shown in Figure 5(b-g). The experiments consist of 2 steps, in the first one the electrode is kept at OCP during 25 s or 30 s for

Ag and Cu respectively (OCP region in Figure 5), and after that a positive current of 150 μA during 50 s for Ag and 400 μA during 95 s for Cu in a 0.1 M $HClO_4$ + 150/100 μM fluorescein for Ag and Cu, respectively. Figure S11 also contains the proposed mechanism to explain the origin of EC-SOERS effect. Figure S11A represents the starting conditions of the system, where photoluminescence spectrum of fluorescein is recorded. As can be seen, no Raman signal of this analyte can be observed, due to the lack of Raman enhancement phenomena. After the electrochemical release of metal cations from the electrode (Figure S11B), these metal cations adsorbed on the crystal structures (AgCl or CuI) on the electrode surface. After that, a complex between the target molecule and the cation absorbed on the nanocrystals is formed. We propose that the formation of this complex could be the responsible for the observed loss of photoluminescence, due to the participation of non-radiative relaxation processes, and for the observed Raman enhancement, due to charge transfers taking place between crystal and molecule (Figure S11C).

Detailed description of parallel absorption UV/Vis and Raman SEC experiments

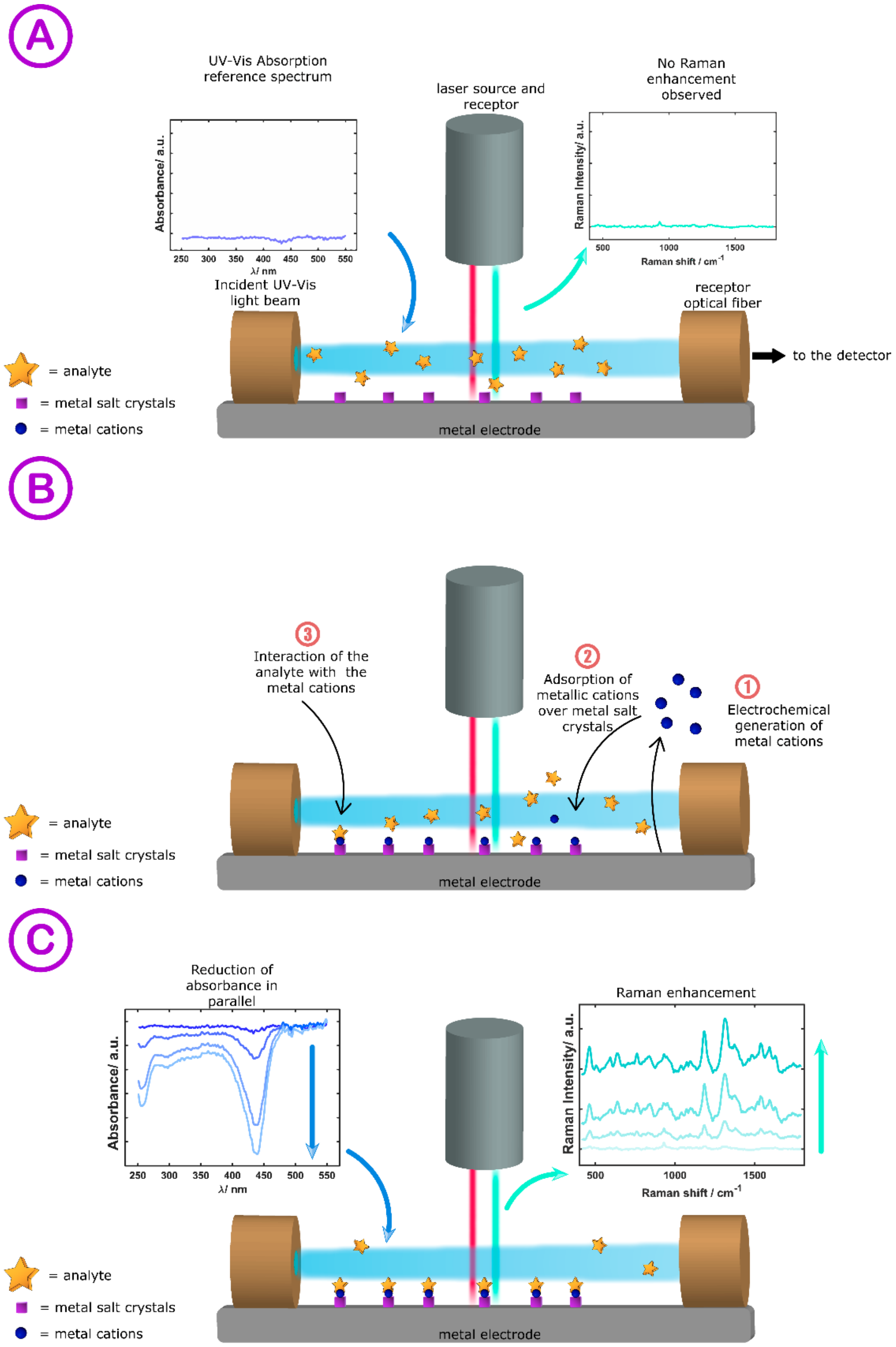

Figure S12. Schematic of the description and interpretation of the experiments shown in Figure 5 (i-n). The schematic shows the proposed evolution of the system during the electrochemical oxidation of a SOERS substrate in this case, Ag/AgCl or Cu/CuI, in presence of a target molecule: (a) Initial system state where no potential is applied to the system; (b) EC-SOERS experiment in which sufficiently anodic potential are applied to partially oxidize the metal surface of the electrode; (c) Interaction between the target molecule and the crystals occurs and changes in both, UV/Vis absorption and Raman signals are observed.

Figure S12 shows a schematic of the experimental setup used to perform parallel UV/Vis absorption and Raman measurements simultaneously during the SEC experiments shown in Figure 5 (i-n). The experiments consist of 2 steps, in the first one the electrode is kept at OCP during 20 s (OCP region in Figure 5), and after that a positive current of 150 µA for Ag and 400 µA for Cu during 55 s in a 0.1 M $HClO_4$ + 150/100 µM fluorescein for Ag and Cu, respectively. Figure S12 also summarizes the proposed mechanism to explain the origin of EC-SOERS. Figure S12A represents the initial conditions of the system, where the UV/Vis absorption spectrum taken as reference (blank) is that of the starting solution. After the electrochemical generation of metal cations (Figure S11B) the metal cations absorb on the metal salt crystalline structures (AgCl o CuI), and the interaction between the analyte and the adsorbed cations takes place (Figure S12C). We propose that these processes generate a gradient in the concentration of analyte in the proximity of the electrode surface, reducing the concentration of molecule in the region sampled by the UV/Vis light beam in parallel configuration with the consequent negative absorption band related to the adsorption of the molecule.

Raman and photoluminescence of Fluorescein in absence of SOERS substrate

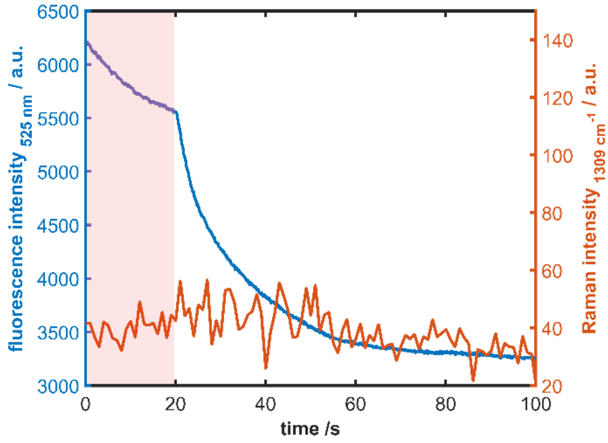

Figure S13. Evolution of photoluminescence emission at 525 nm (blue line) and Raman intensity (orange line) at 1309cm^{-1} of fluorescein during the galvanostatic oxidation of a non-modified Ag-SPE electrode. Electrolytic medium: 150 µM fluorescein + 0.1 M $HClO_4$. Laser source: 785 nm. Excitation source: 405 nm. Current applied: OCP (first 20 s) and 100 µA during the last 80 s.

Figure S13 shows a control experiment that should be compared with the results shown in Figure 5 a-g. In this experiment, the galvanostatic oxidation of a non-modified silver electrode was carried out in the presence of 150 µM fluorescein + 0.1 M $HClO_4$. During the first 20 s of experiment, the electrode is at OCP to observe the natural evolution of the fluorescence signal. It is observed that a natural decay which occurs with time. After 20 s, a constant current of 100 µA was applied. The generation of silver fluorescein in solution leads to the decrease of the fluorescence of fluorescein. Nevertheless, no Raman enhancement is observed in this experiment due to the absence of SOERS substrate, like AgCl. In the absence of this substrate, the Raman signal of silver fluorescein complex is not enhanced. When nanocrystals are present on the electrode surface, Figure 5 in the manuscript, the enhancement of the Raman signal is clearly observed.

Correlation between photoluminescence/absorbance and Raman signal

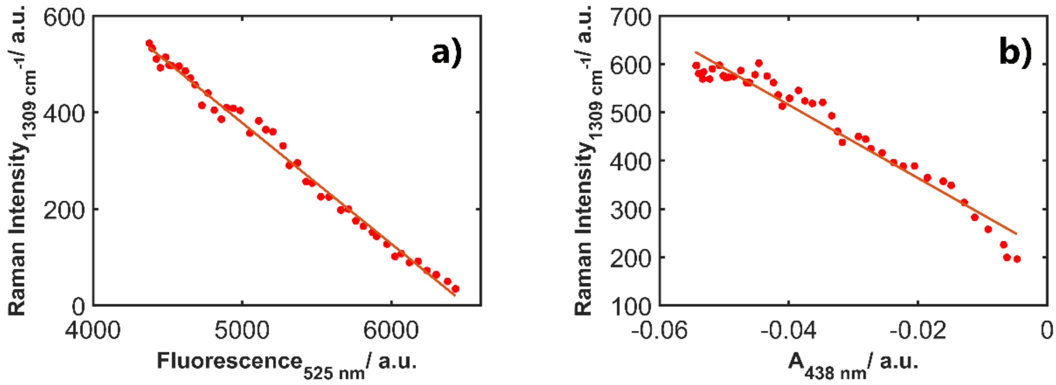

Figure S14. Correlation between (a) Raman Intensity at 1309 cm^{-1} and fluorescence at 525 nm and (b) Raman Intensity at 1309 cm^{-1} and absorbance at 438 nm, corresponding to the experiments shown in Figure 5b and 5i, respectively. Optical signals obtained during the oxidation of the Ag/AgCl electrode in presence of fluorescein. Details of the experimental conditions in Figures 5, S11 and S12.

EC-SOERS response of riboflavin by galvanostatic pulses

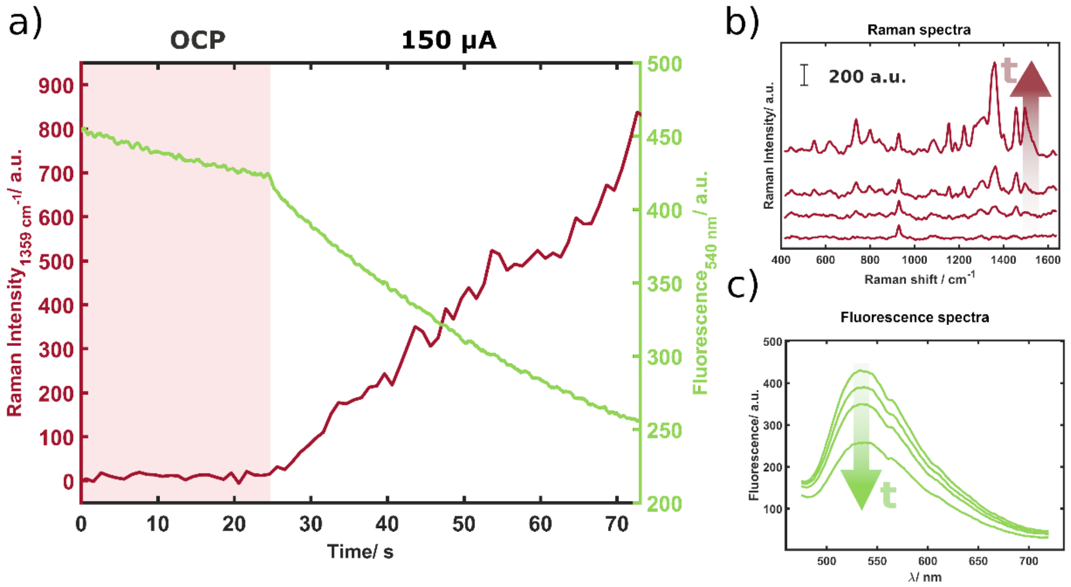

Figure S15. (a) Evolution of the Raman intensity at 1359 cm^{-1} and photoluminescence at 540 nm of riboflavin during the galvanostatic oxidation of a Ag/AgCl SOERS substrate. Evolution of the full Raman (b) and photoluminescence (c) spectra during the experiment. Electrochemical medium: 50 µM Riboflavin + 0.1 M $HClO_4$. Laser source: 785 nm. Excitation source: 385 nm. Current applied: OCP (first 25 s) and 50 µA during the last 45 s.

Figure S15 represents the evolution of photoluminescence and Raman spectra of riboflavin during the galvanostatic oxidation of a Ag/AgCl SOERS substrate. When the electrode is maintained at OCP, the spectra of fluorescence can be clearly observed between 500 and 650 nm, but no appreciable Raman spectra is registered. When a pulse of 50 µA current is applied to the system, a clear and gradual enhancement of the Raman spectra of riboflavin is observed with a simultaneous decrease in the photoluminescence intensity. The fluorescence quenching could be explained by the interaction of riboflavin with Ag^+ cations formed during oxidation of the electrode, a behavior vastly reported previously.[22–25] However, the mere presence of a coordination complex does not explain the Raman enhancement observed, since ordinary mixtures of Ag^+ salts and riboflavin does not generate a visible Raman spectrum at this concentration in absence of AgCl nanocrystals. Therefore, this enhancement is attributed to the interaction of riboflavin and adsorbed Ag^+ on the AgCl nanocrystals surface, which explains the quenching of the photoluminescence and could explain the enhancement of the Raman signals due to a number of phenomena of charge transfer and plasmonic resonances that could take place in the Ag^+/AgCl nanocrystals systems.

Correlation of the AgCl Raman band with the fluorescein Raman band.

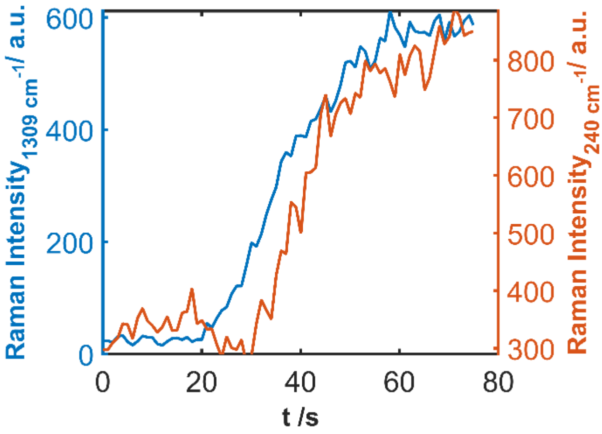

Figure S16. Evolution of the Raman Intensity at 1309 cm^{-1} (blue line) and the Raman Intensity at 240 cm^{-1} (orange line) with time. Raman signals corresponding to the experiments shown in Figure 5i, obtained during the oxidation of the Ag/AgCl electrode in presence of fluorescein. Details of the experimental conditions in Figures 5 and S12.

Correlation between EC-SOERS and Ag^{+} concentration

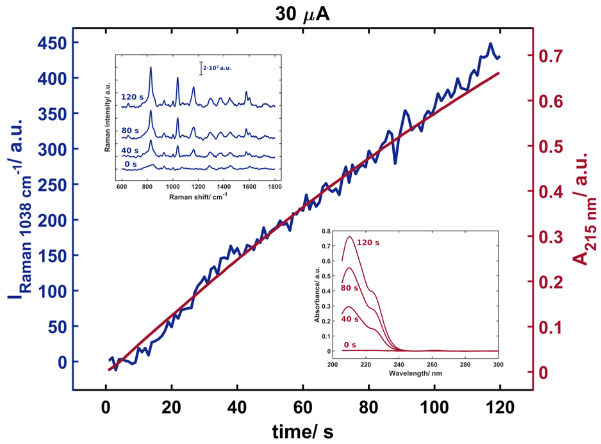

Figure S17. Evolution of the Raman spectra of phthalic acid (inset in left top) and the UV/Vis absorption spectra measured in parallel configuration (inset in right bottom) during the galvanostatic oxidation (30 μA during 120 s) of a Ag/AgCl SOERS substrate. Electrolytic conditions: 0.2 mM phthalic acid + 0.1 M $HClO_4$.

Figure S17 represents both the evolution of the Raman signal of phthalic acid and the evolution of the silver concentration during the galvanostatic oxidation of a silver electrode. In this experiment, we registered simultaneously the Raman response of the electrode and the UV/Vis absorption spectra of the solution close to the electrode, using two optical fibers in parallel configuration. The experimental setup is the same than that shown in Figure S12. This setup allows us to evaluate the species adsorbed on the electrode with Raman spectroscopy and also the species present in the diffusion layer with UV/Vis absorption spectra. When the Ag/AgCl SOERS substrate is oxidized by applying a galvanostatic pulse, a continuous enhancement of the Raman signal related to the phthalic acid is observed. Also, the evolution of an absorption band centered at 215 nm is observed in the UV/Vis spectra, corresponding to the electrogeneration of Ag^{+} cation. A correlation between the Ag^{+} absorption signal and the Raman enhancement of phthalic acid is clearly observed. When a similar experiment is performed on a bare AgSPE (without AgCl crystals), no Raman enhancement of the molecule is observed (data not shown).

Improving the sensitivity of EC-SOERS

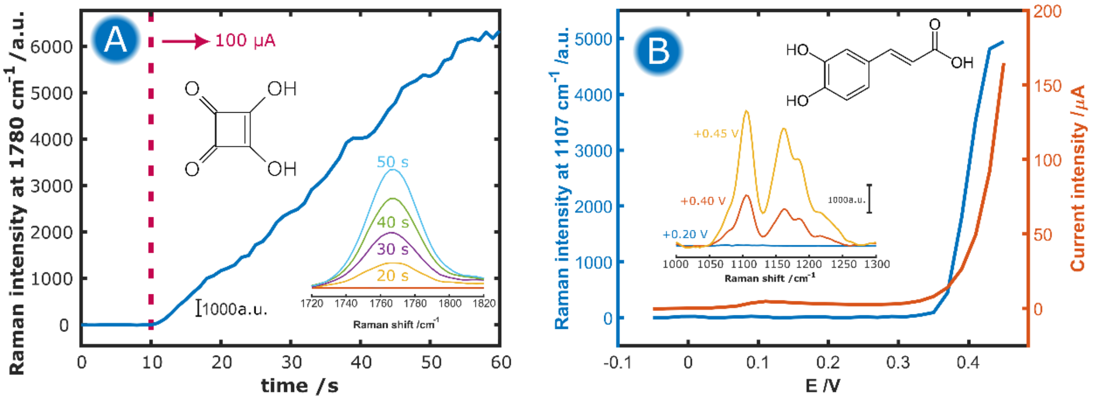

Figure S18. Evolution of the Raman intensity (blue lines) of (A) 50 nM squaric acid and (B) 500 nM caffeic acid during the oxidation of (A) a Ag/AgSCN modified electrode in 0.1 M $HClO_4$ and (B) a bare silver electrode in 1 mM KSCN and 0.1 M $HClO_4$. Oxidation of the Ag/AgSCN electrode in (A) was carried out galvanostatically (open circuit for 10 s, 100 µA for 50 s), meanwhile oxidation of Ag electrode in (B) was carried out by LSV (-0.05 V to +0.45 V vs Ag, 0.02 V s^{-1}). The corresponding LSV (orange line) is plotted in the right axis. Laser power was fixed to 207 mW. Laser source used was 785 nm.

EC-SOERS methodology can be used to achieve great sensitivity in the detection of molecular species, even at nanomolar level. In Figure S18, it is shown 2 different strategies for the nanomolar detection of squaric acid and caffeic acid. In the first strategy, (Figure S18A), it is carried the galvanostatic oxidation of a Ag/AgSCN SOERS substrate in acidic medium. This experiment is analogous to those shown previously, such as Figure S15 or Figure 5 in the main text. Here, the EC-SOERS substrate clearly enhances the Raman signal of squaric acid, even at concentrations as low as 50 nM, demonstrating the sensitivity of the SOERS methodology.

The synthesis of the SOERS substrate and the oxidation of the Ag electrode can be also carried out in one single step. In Figure S18B, it is shown the oxidation of a bare Ag electrode in the presence of KSCN, similar to that shown in Figure S1D. A small anodic signal is observed at +0.15 V, associated with the generation of AgSCN (Figure S2D). The massive oxidation of the electrode to release Ag^+ is observed at +0.35 V, where the Raman enhancement of caffeic acid is observed even at 500 nM.

These experiments demonstrate that really low detection limits can be achieved by different SOERS methodologies. The different strategies shown open new gates for the development of analytical methods based on SOERS/EC-SOERS. The use of one-step methodologies such as the one shown in Figure S18B is truly interesting for the development of simple and reproducible analytical measurements. Meanwhile, the use of different synthetic procedures of SOERS substrates and its posterior characterization opens new possibilities for the design and study of SOERS substrates.

References

(1) Garoz-Ruiz, J.; Heras, A.; Palmero, S.; Colina, A. Development of a Novel Bidimensional Spectroelectrochemistry Cell Using Transfer Single-Walled Carbon Nanotubes Films as Optically Transparent Electrodes. *Anal Chem* **2015**, *87* (12), 6233–6239. https://doi.org/10.1021/acs.analchem.5b00923.

(2) Wonner, K.; Evers, M. V.; Tschulik, K. The Electrochemical Dissolution of Single Silver Nanoparticles Enlightened by Hyperspectral Dark-Field Microscopy. *Electrochim Acta* **2019**, *301*, 458–464. https://doi.org/10.1016/j.electacta.2019.01.129.

(3) Brasiliense, V.; Patel, A. N.; Martinez-Marrades, A.; Shi, J.; Chen, Y.; Combellas, C.; Tessier, G.; Kanoufi, F. Correlated Electrochemical and Optical Detection Reveals the Chemical Reactivity of Individual Silver Nanoparticles. *J Am Chem Soc* **2016**, *138* (10), 3478–3483. https://doi.org/10.1021/jacs.5b13217.

(4) Cooney, R. P.; Reid, E. S.; Fleischmann, M.; Hendra, P. J. Thiocyanate Adsorption and Corrosion at Silver Electrodes. A Raman Spectroscopic Study. *Journal of the Chemical Society, Faraday Transactions 1: Physical Chemistry in Condensed Phases* **1977**, *73*, 1691. https://doi.org/10.1039/f19777301691.

(5) Bron, M.; Holze, R. Cyanate and Thiocyanate Adsorption at Copper and Gold Electrodes as Probed by in Situ Infrared and Surface-Enhanced Raman Spectroscopy. *Journal of Electroanalytical Chemistry* **1995**, *385* (1), 105–113. https://doi.org/10.1016/0022-0728(94)03765-U.

(6) Son, Y.; de Tacconi, N. R.; Rajeshwar, K. Photoelectrochemistry and Raman Spectroelectrochemistry of Cuprous Thiocyanate Films on Copper Electrodes in Acidic Media. *Journal of Electroanalytical Chemistry* **1993**, *345* (1–2), 135–146. https://doi.org/10.1016/0022-0728(93)80474-V.

(7) Perales-Rondon, J. V.; Hernandez, S.; Heras, A.; Colina, A. Effect of Chloride and PH on the Electrochemical Surface Oxidation Enhanced Raman Scattering. *Applied Surface Science* **2019**, *473* (December 2018), 366–372. https://doi.org/10.1016/j.apsusc.2018.12.148.

(8) Shafir, I.; Nagli, L.; Katzir, A. Raman Spectroscopy of Rare Earth Doped Silver Halide Crystals. *Appl Phys Lett* **2009**, *94* (23), 1–4. https://doi.org/10.1063/1.3148707.

(9) Chen, Y.; Zhao, C.; Ma, S.; Xing, P.; Hu, X.; Wu, Y.; He, Y. Fabrication of a Z-Scheme AgBr/Bi4O5Br2 Nanocomposite and Its High Efficiency in Photocatalytic N2 Fixation and Dye Degradation. *Inorg Chem Front* **2019**, *6* (11), 3083–3092. https://doi.org/10.1039/c9qi00782b.

(10) Hernandez, S.; Perales-Rondon, J. V.; Heras, A.; Colina, A. Simultaneous Raman and Reflection UV/Vis Absorption Spectroelectrochemistry. *Nano Res* **2022**. 15 (6): 5340−5346. https://doi.org/10.1007/s12274-022-4137-5.

(11) Fleischmann, M.; Graves, P. R.; Robinson, J. The Raman Spectroscopy of the Ferricyanide/Ferrocyanide System at Gold, β-Palladium Hydride and Platinum Electrodes. *Journal of Electroanalytical Chemistry* **1985**, *182* (1), 87–98. https://doi.org/10.1016/0368-1874(85)85442-3.

(12) Cooney, R. P.; Reid, E. S.; Fleischmann, M.; Hendra, P. J. Thiocyanate Adsorption and Corrosion at Silver Electrodes. A Raman Spectroscopic Study. *Journal of the Chemical*

Society, Faraday Transactions 1: Physical Chemistry in Condensed Phases **1977**, *73*, 1691. https://doi.org/10.1039/f19777301691.

(13) Kennedy, J. V.; Murmu, P. P.; Karthik, V.; Liu, Z.; Jovic, V.; Mori, T.; Yang, W. L.; Smith, K. E. Influence of Carrier Density and Energy Barrier Scattering on a High Seebeck Coefficient and Power Factor in Transparent Thermoelectric Copper Iodide. *ACS Appl Energy Mater* **2020**, *3* (10), 10037–10044. https://doi.org/10.1021/acsaem.0c01724.

(14) Utrera-Melero, R.; Huitorel, B.; Cordier, M.; Mevellec, J. Y.; Massuyeau, F.; Latouche, C.; Martineau-Corcos, C.; Perruchas, S. Combining Theory and Experiment to Get Insight into the Amorphous Phase of Luminescent Mechanochromic Copper Iodide Clusters. *Inorg Chem* **2020**, *59* (18), 13607–13620. https://doi.org/10.1021/acs.inorgchem.0c01967.

(15) Aldakov, D.; Chappaz-Gillot, C.; Salazar, R.; Delaye, V.; Welsby, K. A.; Ivanova, V.; Dunstan, P. R. Properties of Electrodeposited CuSCN 2D Layers and Nanowires Influenced by Their Mixed Domain Structure. *The Journal of Physical Chemistry C* **2014**, *118* (29), 16095–16103. https://doi.org/10.1021/jp412499f.

(16) Yang, Z.; Wu, B.; Zhai, C.; Niu, S.; Sun, B.; Dang, L.; Gu, C.; Qi, X.; Tian, Y.; Li, J.; Ma, S.; Yao, M. Pressure-Dependent Structural and Band Gap Tuning of Semiconductor Copper(I) Thiocyanate (CuSCN). *Inorg Chem* **2022**, No. I. https://doi.org/10.1021/acs.inorgchem.2c03024.

(17) Wang, L.; Roitberg, A.; Meuse, C.; Gaigalas, A. K. Raman and FTIR Spectroscopies of Fluorescein in Solutions. *Spectrochim Acta A Mol Biomol Spectrosc* **2001**, *57* (9), 1781–1791. https://doi.org/10.1016/S1386-1425(01)00408-5.

(18) Wang, L.; Roitberg, A.; Meuse, C.; Gaigalas, A. K. Raman and FTIR Spectroscopies of Fluorescein in Solutions. *Spectrochim Acta A Mol Biomol Spectrosc* **2001**, *57* (9), 1781–1791. https://doi.org/10.1016/S1386-1425(01)00408-5.

(19) Sardo, M.; Ruano, C.; Castro, J. L.; López-Tocón, I.; Soto, J.; Ribeiro-Claro, P.; Otero, J. C. Surface-Enhanced Raman Scattering of 5-Fluorouracil Adsorbed on Silver Nanostructures. *Physical Chemistry Chemical Physics* **2009**, *11* (34), 7437. https://doi.org/10.1039/b903823j.

(20) Alessandri, I.; Lombardi, J. R. Enhanced Raman Scattering with Dielectrics. *Chem Rev* **2016**, *116* (24), 14921–14981. https://doi.org/10.1021/acs.chemrev.6b00365.

(21) Kuhlman, A. K.; Zayak, A. T. Revealing Interaction of Organic Adsorbates with Semiconductor Surfaces Using Chemically Enhanced Raman. *J Phys Chem Lett* **2014**, *5* (6), 964–968. https://doi.org/10.1021/jz500024x.

(22) Fabbrizzi, L.; Licchelli, M.; Pallavicini, P.; Sacchi, D.; Taglietti, A. Sensing of Transition Metals through Fluorescence Quenching or Enhancement. A Review. *Analyst* **1996**, *121* (12), 1763. https://doi.org/10.1039/an9962101763.

(23) Shamsipur, M.; Alizadeh, K.; Hosseini, M.; Caltagirone, C.; Lippolis, V. A Selective Optode Membrane for Silver Ion Based on Fluorescence Quenching of the Dansylamidopropyl Pendant Arm Derivative of 1-Aza-4,7,10-Trithiacyclododecane ([12]AneNS3). *Sens Actuators B Chem* **2006**, *113* (2), 892–899. https://doi.org/10.1016/j.snb.2005.03.117.

(24) Tan, S. S.; Kim, S. J.; Kool, E. T. Differentiating between Fluorescence-Quenching Metal Ions with Polyfluorophore Sensors Built on a DNA Backbone. *J Am Chem Soc* **2011**, *133* (8), 2664–2671. https://doi.org/10.1021/ja109561e.

(25) Sharma, A. K.; Priya; Kaith, B. S.; Isha; Singh, A.; Chandel, K.; Vipula. Riboflavin Functionalized Dextrin-Sodium Alginate Based Fluorescent Sensor: Detoxification of Cu 2+ and Ni 2+ Ions. *ACS Appl Polym Mater* **2019**, *1* (11), 3084–3094. https://doi.org/10.1021/acsapm.9b00724.
